# Tripeptides conjugated with thiosemicarbazones: new inhibitors of tyrosinase for cosmeceutical use

**DOI:** 10.1080/14756366.2023.2193676

**Published:** 2023-05-05

**Authors:** Patrycja Ledwoń, Waldemar Goldeman, Katarzyna Hałdys, Michał Jewgiński, Greta Calamai, Joanna Rossowska, Anna Maria Papini, Paolo Rovero, Rafał Latajka

**Affiliations:** a Department of Bioorganic Chemistry, Faculty of Chemistry, Wroclaw University of Science and Technology, Wrocław, Poland; b Interdepartmental Research Unit of Peptide and Protein Chemistry and Biology, Department of Neurosciences, Psychology, Drug Research and Child Health Section of Pharmaceutical Sciences and Nutraceutics, University of Florence, Sesto Fiorentino, Italy; c Department of Organic and Medicinal Chemistry, Faculty of Chemistry, Wroclaw University of Science and Technology, Wrocław, Poland; d Ludwik Hirszfeld Institute of Immunology and Experimental Therapy, Polish Academy of Science, Wrocław, Poland; e Interdepartmental Research Unit of Peptide and Protein Chemistry and Biology, Department of Chemistry “Ugo Schiff”, University of Florence, Sesto Fiorentino, Italy

**Keywords:** Tyrosinase, thiosemicarbazones, peptide conjugates, cosmeceuticals melanogenesis

## Abstract

The development of skin-care products is recently growing. Cosmetic formulas containing active ingredients with proven efficacy, namely cosmeceuticals, are based on various compounds, including peptides. Different whitening agents featuring anti-tyrosinase activity have been applied in the cosmeceutical field. Despite their availability, their applicability is often limited due to several drawbacks including toxicity, lack of stability, and other factors. In this work, we present the inhibitory effect on diphenolase activity of thiosemicarbazone (TSC)-peptide conjugates. Tripeptides FFY, FWY, and FYY were conjugated with three TSCs bearing one or two aromatic rings *via* amide bond formation in a solid phase. Compounds were then examined as tyrosinase and melanogenesis inhibitors in murine melanoma B16F0 cell line, followed by the cytotoxicity assays of these cells. *In silico* investigations explained the differences in the activity, observed among tested compounds. Mushroom tyrosinase was inhibited by TSC**1**-conjugates at micromolar level, with IC_50_ lower than this for kojic acid, a widely used reference compound. Up to now, this is the first report regarding thiosemicarbazones conjugated with tripeptides, synthesised for the purpose of tyrosinase inhibition.

## Graphical abstract

**Figure:**
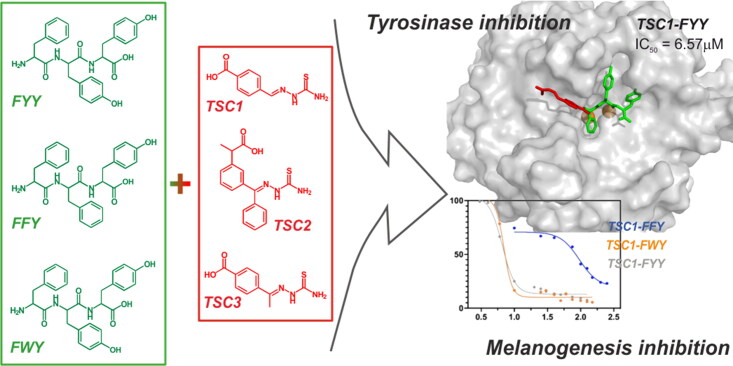

## Introduction

The interest in the development of skin whitening agents is growing. A report on skin lightening products market published by Grand View Research shows that the global market of skin-lightening products was valued at USD 9.96 billion in 2021 and is expected to expand by 5.5% at a compound annual growth rate (CAGR) from 2022 to 2030.^1^

Melanin is the pigment responsible for skin and hair colour in mammals. It also absorbs ultraviolet radiation protecting the skin from damage.^2^ The key enzyme in melanin production is tyrosinase (EC 1.14.18.1) which is able to oxidise both monophenols to *o*-quinones and *o*-diphenols to *o*-quinones in two different steps during melanogenesis.^3^ Two copper ions in the active site of tyrosinase are important for catalytic activity.^4^ Overproduction, accumulation, and uneven distribution of melanin in mammalian skin lead to numerous pigmentary disorders, involving freckles, age spots, melasma, sites of actinic damage^5^ or even malignant melanoma, a type of skin cancer that develops from pigment-producing cells (melanocytes).^6^ Therefore, one of the most common approaches for skin pigmentation control involves the inhibition of tyrosinase, the key enzyme in melanin production. Various tyrosinase inhibitors of both natural and synthetic sources have been already discussed in some relevant reviews.^7–11^

Despite the availability of a great number of compounds revealing anti-tyrosinase activity, their application in the cosmetic industry is very limited due to several drawbacks including toxicity, lack of stability, off-odours, off-flavours, and economic sustainability. Hydroquinone and kojic acid,^12^ known components of anti-pigmentation cosmetics, have been reported to be unsafe in concentrations required for a substantial skin-lightening effect.^13^^,^^14^ Therefore, there is a need to search for novel effective and safe tyrosinase inhibitors.

Peptides are rapidly expanding as cosmeceutical active ingredients.^15^^,^^16^ Due to their safety, tolerability decreased side effects, and low toxicity they are considered attractive ingredients for many skin-related indications.^17–20^ The latest review on peptides as tyrosinase inhibitors point out that peptides are able to efficiently form hydrogen and hydrophobic bonds, as well as interact with copper in the active site of the enzyme.^21^ It was proven that the presence of hydrophobic amino acids at the N- and C-termini of the peptide chains are involved in additional non-covalent interactions within copper-containing active sites of tyrosinase.^22^ Tyrosine located at the C-terminus of the peptide sequences enhanced the overall inhibitory effect and this phenomenon was confirmed by molecular modelling.^23^ Moreover, only short sequences, i.e. di-, tri-, and tetrapeptides were found to be capable of binding histidine residues in the active site.^22^

Both short and long peptides can be conjugated with small organic molecules, hence acting in different modes. The peptide moiety can be the active ingredient, but it can also be a carrier or a stabilising agent.^16^^,^^24^^,^^25^ Recently, the use of peptide conjugates has increased, with particular attention on pharmaceutical applications, as demonstrated by ConjuPepDB, a database of peptide-drug conjugates, recently published by Balogh et al.^26^^,^^27^ In most cases, the peptide is not mandatory for the activity, but it enhances the overall efficacy, for example, by additional binding specificity.^25^^,^^28^^,^^29^ Various chemical linkages are used to perform the conjugation, the most frequently utilised are amide, sulphonamide, disulphide, glucuronide, carbamate, ether, and ester bonds.^26^^,^^28^ It was reported that tripeptides conjugated with a small organic molecule, i.e. kojic acid,^30^^,^^31^ mimosine,^32^ kaffeic acid,^33^ are more potent tyrosinase inhibitors than the original organic molecule or the tripeptide alone. For both conjugates, the amino acid sequences in kojic acid-tripeptide^30^ and mimosine-tripeptide^32^ of the most effective tripeptides were consistent. Phenylalanine was the first amino acid in the sequence conjugated with the organic molecule, tyrosine was consequently located at the C-terminus, while one of the amino acids containing an aromatic residue (Phe, Tyr, or Trp) was located in the middle of the chain.

Thiosemicarbazones (TSCs) represent a versatile class of ligands. They possess multiple biological, pharmacological, and therapeutic properties that make them effective antibacterial,^34^ antifungal,^35^ antiviral,^36^ and anticancer^37^ agents. Over the past ten years, numerous aromatic thiosemicarbazone derivatives endowed with potent anti-tyrosinase activity have been reported.^38–44^ Possibly, the aromatic residue may interact with the hydrophobic cavity of the enzyme *via* van der Waals interactions, and the N-N-S tridentate coordination scaffold may act as a chelator of catalytic copper ions.^45^ In our previous papers we reported that *para*-substituted acetophenone TSC derivatives are more potent tyrosinase inhibitors than their analogues substituted at ortho or meta positions, and in many cases than unsubstituted ones^46^^,^.^47^ Similar observations were made for benzaldehyde TSC.

The main purpose of the present work is to evaluate the inhibitory activity of a series of TSC-peptide conjugates on diphenolase activity of tyrosinase. Nine tripeptides conjugated with TSCs bearing one or two aromatic rings have been synthesised. The effect of these conjugates on the activity of mushroom tyrosinase has been investigated. The nature of tyrosinase-inhibitor interactions was explained with molecular docking. We finally studied the inhibition of melanin production of the new thiosemicarbazone conjugates in murine melanoma B16F0 cell line and their cytotoxicity in the same cells. The above-mentioned results may be useful for designing novel tyrosinase inhibitors with cosmeceutical relevance, possessing skin-whitening properties.

## Materials and methods

### Synthesis, purification, analysis

#### Materials

For the synthesis of TSCs, all solvents were of commercial quality and purchased from a local supplier (Avantor). Thiosemicarbazide (catalogue nr T33405); racemic ketoprofen (catalogue nr K1751); 4-acetylbenzoic acid (catalogue nr 177458); 4-formylbenzoic acid (catalogue nr 124915); and *p*-toluenesulfonic acid monohydrate (catalogue nr T35920) were purchased from Merck (Darmstadt, Germany).

For peptide synthesis, all Fmoc-protected amino acids Fmoc-Phe-OH, Fmoc-Tyr(*t*Bu)-OH, and Fmoc-Trp(Boc)-OH were purchased from Iris Biotech GmbH (Marktredwitz, Germany). Peptide synthesis grade *N,N*′-Dimethylformamide (DMF) and acetonitrile (ACN) were purchased from Carlo Erba (Milano, Italy). Dichloromethane (DCM), trifluoroacetic acid (TFA), triisopropylsilane (TIS), methanol (MeOH), piperidine, *N,N*′-Diisopropylethylamine (DIPEA), *N*-Methylmorpholine (NMM), 1,2-Ethanedithiol (EDT), and acetic anhydride were purchased from Sigma-Aldrich (Milano, Italy). Fmoc-Tyr(*t*Bu)-Wang resin (100–200 mesh, loading: 0.70 mmol/g) was purchased from Novabiochem (Merck, Darmstadt, Germany).

#### Purification of peptides and TSC-conjugates

Crude peptides and TSC-conjugates were purified by Reverse-Phase Flash Liquid Chromatography (RP-HPLC) on Isolera One Flash Chromatography (Biotage, Uppsala, Sweden) using a SNAP Ultra C18 column (40 g) at 20 ml/min flow. Eluent systems: 0.1% TFA in H_2_O (A), 0.1% TFA in ACN (B). Due to the poor solubility in water of final compounds, the samples for purification were dissolved in the mixture of solvents A and B at the initial percentage of the purification gradient and treated with ultrasounds. The linear gradient applied for each purification was: 35–65% B in A in 30 min.

#### LC-MS analysis

HPLC-MS experiments of peptides and TSC-conjugates were performed using *Alliance Chromatography model 2695* (Waters, Milford, U.S.A.) with a Phenomenex Kinetex C18 column (2.6 μm, 3.0 × 100 mm), flow 0.6 ml/min, coupled to a single quadrupole ESI-MS (Micromass Z*Q*) at 6 ml/min of: 0.1% TFA in H_2_O (A), 0.1% TFA in 84% ACN/H_2_O (B), λ = 254 nm, gradient: 30–90% B in 5 min (for the conjugates) and 20–70% (for control tripeptides), injection volume: 10 µL. High-resolution Mass Spectra of thiosemicarbazones **1–3** were recorded on an LCT Premier XE Waters apparatus, in ESI + mode.

#### NMR analysis

The ^1^H and ^13^C NMR spectra were recorded on a Jeol ECZ 400S spectrometer in DMSO-*d*_6_ as solvent. Chemical shifts (δ) are given in parts per million (ppm) relative to solvent signals (2.50 ppm and 39.52 ppm in ^1^H NMR and in ^13^C NMR spectra, respectively). Multiplicity is reported as follows: s = singlet, d = doublet, t = triplet, q = quartet, quint = quintet, sext = sextet, hept = heptet, m = multiplet, br = broad, bs = broad singlet.

### Synthesis of thiosemicarbazones

Thiosemicarbazones **1–3** were prepared according to the procedure described previously in our papers.^46–48^ Detailed protocol and NMR data of the obtained products are reported in the Supplementary Information.

### Solid-phase peptide synthesis

Peptides Ac-FXaaY-OH (where Xaa = F,W,Y) were synthesised manually in solid-phase following the Fmoc/*t*Bu orthogonal protection strategy as described elsewhere.^49^^,^^50^ Detailed protocol, chromatograms, and mass spectra of the obtained products are reported in the Supplementary Information.

### Tripeptide conjugates synthesis

Conjugates of peptides and TSCs were synthesised according to the procedure described for the peptides. TSCs were dissolved in DMF and coupled to the free N-terminus peptide chain after Fmoc-deprotection, following the standard coupling protocol (double coupling with 1.5 eq.). TSCs, well soluble in DMF, do not require any particular treatment and the conjugation undergoes easily. Chromatograms and mass spectra of the obtained products are reported in the Supplementary Information.

### Tyrosinase inhibition assay

The tyrosinase enzymatic assay was performed according to the protocol described previously in our papers^46–48^ using L-3,4-dihydroxyphenylalanine (L-DOPA) (Sigma-Aldrich, Germany) as a substrate. L-DOPA was dissolved in an aqueous 0.15 mM phosphoric(V) acid solution, preventing its oxidation. The final L-DOPA concentration for the assay was 5 mM. TSCs were dissolved in DMSO, achieving the concentration of stock solutions of 10 mM. TSC-conjugates and unmodified peptides were dissolved in a solution of DMSO/sodium phosphate buffer (1:9, 0.1 M, pH 6.8; assay buffer) (v/v), achieving the concentration of stock solutions of 1 mM. All compounds were then diluted in the assay buffer to test concentrations (DMSO concentrations in final reaction mixtures did not exceed 1% volume). Tyrosinase powder (Sigma-Aldrich, Germany) was dissolved in the assay buffer to obtain a concentration of 50,000 units/mL. This stock solution was divided into 50 μL aliquots, each one containing 2500 units of tyrosinase, and stored at −80 °C. Shortly before the assay, one of the aliquots was reconstituted in the assay buffer up to 3000 μL. First, 10 μL of the diluted enzyme solution was preincubated with samples at various concentrations for 5 min at 25 °C. Afterwards, 20 μL of L-DOPA solution was added. The reaction was immediately monitored by measuring the change in absorbance of the colour product (dopachrome) for 10 min in kinetic mode with 5 s intervals (λ = 475 nm, 25 °C) using *Molecular Devices SpectraMax Plus 384 Microplate Reader*. The enzyme control (EC) sample did not contain the inhibitor; the blank sample did not contain either the inhibitor or the substrate. Kojic acid (Sigma-Aldrich, Germany), as a positive control, was treated as the other inhibitors.

### Cell proliferation assay

B16F0 murine melanoma cell line was cultured in DMEM (Biowest, Nuaillé, France) medium supplemented with 100 U/ml streptomycin (Sigma-Aldrich, Taufkirchen, Germany), 100 U/ml penicillin (Sigma-Aldrich, Taufkirchen, Germany), and 10% foetal bovine serum (Sigma-Aldrich, Taufkirchen, Germany). Cells were maintained in 5% CO_2_ at 37 °C. Cells viability was estimated by MTT colorimetric assay as described by Bellei et al.^51^ B16 cells were seeded into 96-well plate (4 × 103 cells/well) and incubated in the presence of the investigated compounds at various concentrations (1000, 400, 100, 40, 10, 1, 0.1 μM) for 48 h. Kojic acid and DMSO were used as controls. MTT was added into wells (0.625 mg/ml, Sigma-Aldrich, Taufkirchen, Germany) for the last 4 h. Then lysing buffer was added to dissolve insoluble formazan. The absorbance was measured (λ = 570 nm) using Thermo Lab Multiscan RC microplate reader.

### Measurement of melanin production

Measurement of melanin production in B16F0 murine melanoma cell line was performed as described by Bellei et al.^51^ The cells were seeded into 96-well plate (5 × 103 cells/well) and stimulated with α-MSH (100 nM, Sigma-Aldrich, Taufkirchen, Germany). After 24 h cells were treated with the investigated compounds as well as DMSO and kojic acid at a final concentration of 100, 40, 10, 4, and 1 μM. After 48 h of incubation with inhibitors, melanin production was measured by evaluating the absorbance (λ = 405 nm) using a microplate reader.

### Molecular modelling

Before the simulation of the docking process, ligand structures were prepared with *Discovery Studio Client.* The structures of all the ligands were optimised in the program *Gaussian16* at the B3LYP/6-311g (d,p).^52^ During the structure optimisation, solvent model PCM was applied. The crystal structure of tyrosinase from *Agaricus bisporus* was obtained from the RCSB Protein Data Bank (ID: 2Y9X).^53^ The ligand, besides copper ions, was removed from the crystal structure of the enzyme. Lacking protons and charges were added to the protein using the *H++* serve according to pH value 6.8^54^.^55^ The active site sphere was selected based on the location of tropolone with a radius 18Ang. Molecular docking in the defined active site of tyrosinase was carried out using *GOLD* *Algorithm* (2021.3.0 version, CCDC, Cambridge, United Kingdom) using the ChemPLP scoring function. The docking using genetic algorithm used default settings (population size: 100, selection pressure: 1.1; the number of operations: 100000; the number of islands: 5; niche size: 2; crossover frequency: 95; mutation frequency: 95; migration frequency: 10). For each inhibitor, ten distances were obtained. Analysis of inhibitor–enzyme interactions of docked molecules was performed with *Discovery Studio Visualiser 5* (Dassault Systemes BIOVIA, U.S.A.).

## Results and discussion

### Design of the new peptide conjugates

**Figure 1.**
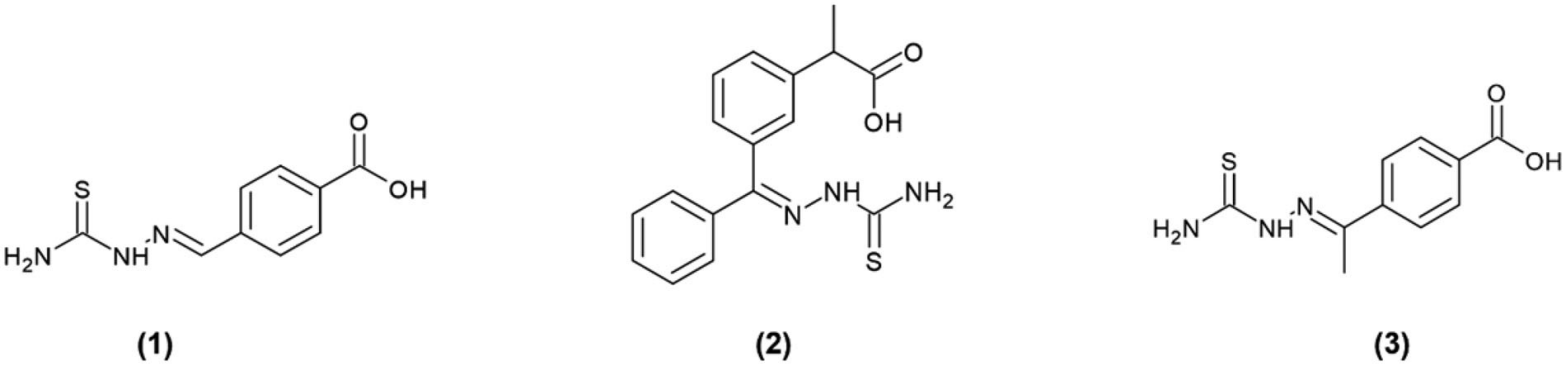
Thiosemicarbazones **1–3** with a free and reactive carboxyl group, available for peptide conjugation.

According to our previous findings, the sulphur atom in the thiourea moiety interacts with copper ions in the active site of tyrosinase, and the aromatic residue is responsible for the hydrophobic interactions.^46^^,^^47^ It may be assumed that the preferable position for the peptide chain attachment should not be located in the proximity of the thiourea group. Therefore, we synthesised different thiosemicarbazones possessing free -COOH group (Figure 1). Compounds **1** and **3**, with the carboxyl group at position para of the aromatic ring, differed from each other by one additional methyl group. TSC **2**, bearing a second branched phenyl ring, was included to evaluate the influence of the presence of two aromatic moieties on the inhibitory properties of the final product. Thus, peptide sequences treated as bulky substituents would have been located in the most convenient position distanced from the thiourea group of each TSC.

As mentioned in the introduction, the peptide sequences chosen for conjugation were selected from the literature, where it was reported that the preferable amino acids should be aromatic and hydrophobic (Phe, Tyr, Trp), and Phe residue should be located at the N-terminus. Thus, we considered the conjugation with three different tripeptides: Phe-Trp-Tyr (FWY), Phe-Tyr-Tyr (FYY), and Phe-Phe-Tyr (FFY).

### Synthesis of thiosemicarbazones

**Scheme 1.**
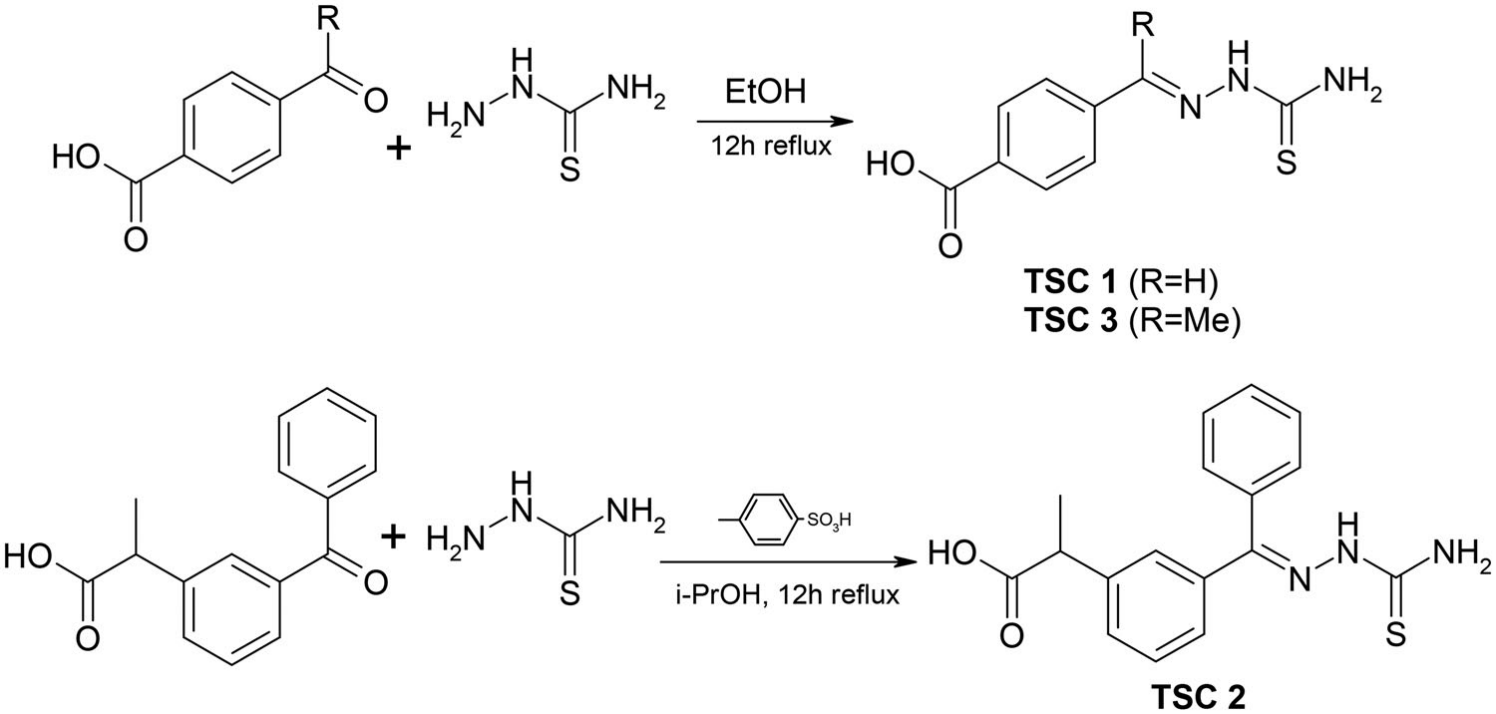
Synthesis of the thiosemicarbazones TSC **1** and TSC **3** (top) and TSC **2** (bottom).

Synthesis of all thiosemicarbazones (TSCs **1–3**) was performed according to the procedure described in detail in our previous papers.^46–48^ As shown in Scheme 1, TSC **1–3** were prepared by the reaction of the appropriate carbonyl precursor with thiosemicarbazide.

Thiosemicarbazones TSC **1** and TSC **3** were obtained by heating stoichiometric amount of thiosemicarbazide and 4-formylbenzoic acid or 4-acetylbenzoic acid in ethanol without any acid catalyst. The preparation of thiosemicarbazone of ketoprofen, TSC **2**, required the use of *p*-toluenesulfonic acid as an acid catalyst and an excess of thiosemicarbazide. TSC **1** and **3** were obtained as single *E* isomers, as confirmed on the basis of the chemical shift of the signal of N^2^H proton (C = N-N*H*-) in the ^1^H NMR spectrum. In the case of the thiosemicarbazones of aromatic aldehydes, the mentioned signal usually appears over 11 ppm^56^^,^,^57^ while the thiosemicarbazones of aryl alkyl ketones give this signal at about 10.2 to 10.6 ppm.^40^^,^^57^^,^^58^ In the case of TSC **1** and **3**, the N^2^H signals were observed at 11.53 and 10.30 ppm, respectively. Since the starting ketoprofen was a racemic mixture, TSC **2** could theoretically exist as a mixture of two pairs of isomers, i.e. R/S mixture of *E* isomer and R/S mixture of *Z* isomer. Thiosemicarbazone TSC **2** gives a ^1^H NMR spectrum with two sets of signals in the 45:55 ratio (based on the integration of the quartettes of CH groups), confirming that TSC **2** was obtained as a nearly equimolar mixture of a racemic mixture of *E* and *Z* isomers.

### Synthesis of peptides and conjugates

Peptides were synthesised in the solid phase according to the standard orthogonal protection Fmoc/*t*Bu strategy, following the procedure described in the *Materials and methods* section. Briefly, all conjugates were obtained by coupling the corresponding TSC to the peptide sequence in N-terminal position *via* amide bond formation. The conjugation was performed in the solid phase after Fmoc-deprotection of the N-terminal position of the tripeptide. Cleavage of the final products, both unmodified sequences and conjugates, was performed in acidic conditions with trifluoroacetic acid (TFA). The conjugated thiosemicarbazones were resistant to both basic and acidic conditions and displayed good solubility in DMF. Peptides and the corresponding conjugates were purified by HPLC and lyophilised for further enzymatic assays. Analytical data (chromatograms and mass spectra) of the synthesised compounds are reported in the Supporting Information.

### Tyrosinase inhibition assay

The screening of tyrosinase activity of the synthetic compounds was performed according to the procedure previously reported in our articles.^46–48^ As reported in most of the research dealing with tyrosinase inhibition, we employed mushroom tyrosinase, due to the lack of information and the limited availability of human tyrosinase.^59^^,^^60^ All the compounds were evaluated at different concentrations after incubation with tyrosinase in solution for 5 min at room temperature. Afterwards, the substrate L-DOPA was added to start the enzymatic reaction. The absorbance provided by the colourful derivative, dopachrome, was measured every 5 s for 10 min in order to monitor the progress of the reaction over time. The percentage of tyrosinase activity was calculated by the formula **(1)**, where: *Sample_slope_* – is the slope value of linear fragment of the absorbance observed for a sample in the presence of the inhibitor; *EC_slope_* – is the slope value of a sample without inhibitor (enzyme control; EC).

(1) $$\%\;\text{of}\;ENZ_{\mathrm{activity}}=\frac{\mathrm{Sampl}e_{\mathrm{slope}}}{EC_{\mathrm{slope}}}\times 100$$

**Figure 2.**
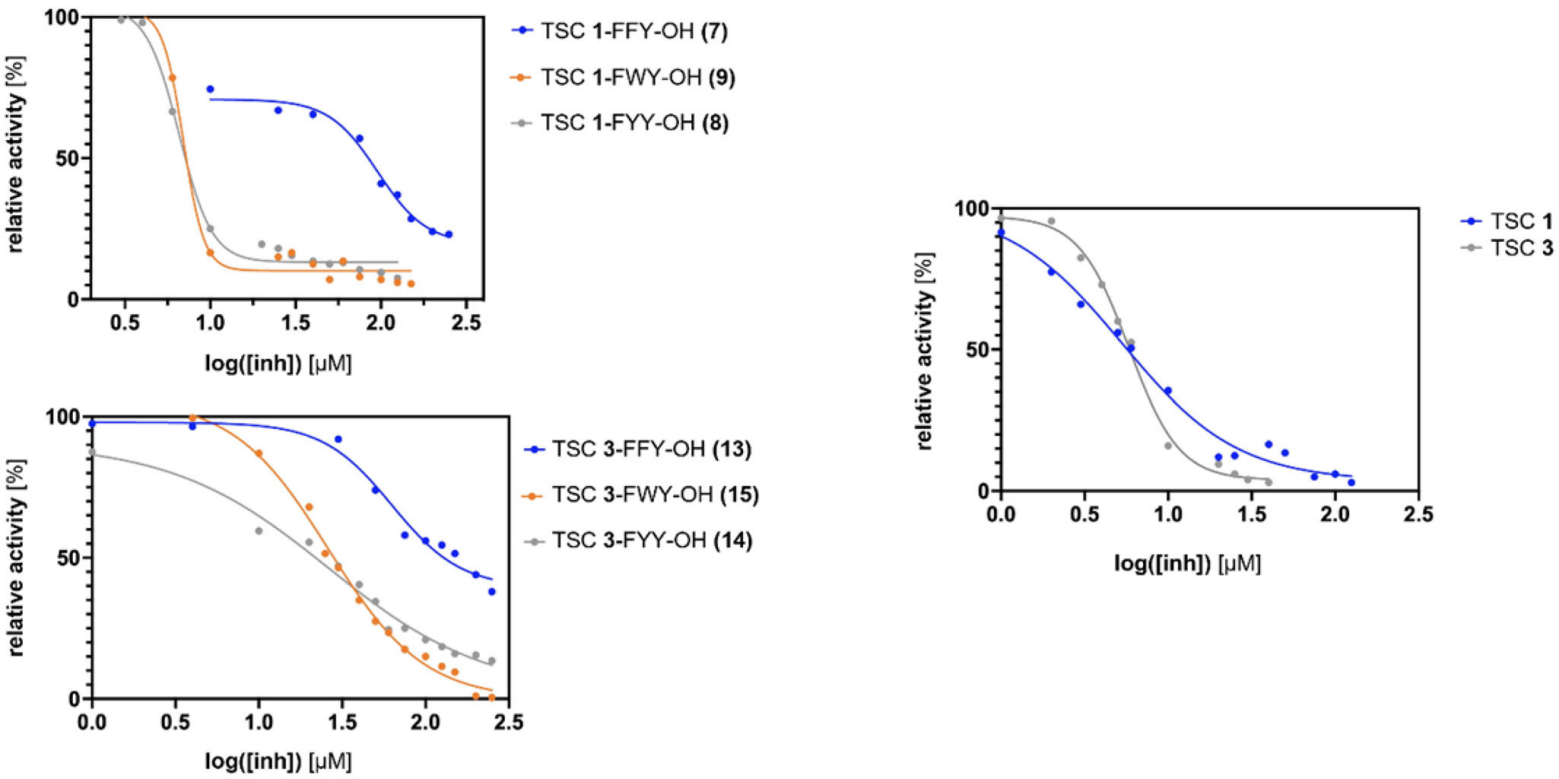
Plots with results of tyrosinase inhibition assay. TSC **1** and **3**, and their tripeptide conjugates **7–9** and **13–15** were found to be the most active inhibitors of tyrosinase among the tested group (IC_50_ representation – relative activity in % *versus* log[inh] in μM).^61^

The effect on the diphenolase activity of the enzyme was expressed as IC_50_ (Figure 2 and Table 1) using *GraphPad Prism* software.^61^ All TSCs and the tripeptide-conjugates demonstrated dose-dependent inhibitory activity. Significant differences between TSCs **1**, **2**, and **3** were observed. The bulkiest TSC **2** acted as the worst tyrosinase inhibitor among the three tested thiosemicarbazones. It provided a maximum of 34% of enzyme inhibition at 150 μM concentration. At higher concentrations of TSC **2** no further decrease of the tyrosinase activity was observed. On the other hand, the two structurally similar TSC **1** and **3** demonstrated comparable inhibition at micromolar level (IC_50_ 5.3 and 5.8 μM, respectively). Moving forward to peptides and their conjugates with TSCs, firstly we analysed unmodified peptides **4–6**. In the tested range of concentrations from 0 to 150 μM, unmodified peptides did not show any significant inhibitory activity (max. observed 5% of inhibition). In accordance with the results obtained for TSCs, the TSC **2-**tripeptide conjugates **10**–**12** were poor inhibitors of tyrosinase (max. 50% of inhibition for C_M_=250 μM). In contrast, the conjugates with TSC **1** generally maintained the inhibitory activity of the thiosemicarbazone (IC_50_=6.57 and 6.66 μM), except for FFY sequence (IC_50_=95.56 ± 25 μM). The tripeptide conjugates with TSC **3**, i.e. **13**–**15**, displayed a decrease in inhibitory activity compared to the unconjugated TSC molecule. Once again, FYY- and FWY-tripeptide derivatives (**14** and **12**) displayed similar tyrosinase inhibition (IC_50_=26 and 25 μM, respectively), but that was remarkably worse in the case of the FFY-conjugate **13** (62% of inhibition for 250 μM).

**Table 1.** Results of tyrosinase inhibition assay.

Compound		Obtained values (*n* = 3)	
		*IC_50_ [μM]*	*The maximum percentage of inhibition reached for poor inhibitors*
Kojic acid		20	–
1	TSC**1**	5.3 ± 2.2	–
2	TSC**2**	–	34% (150 μM)
3	TSC**3**	5.8 ± 0.5	–
4	Ac-FFY-OH	no significant inhibition was observed in the tested range of concentrations	
5	Ac-FYY-OH		
6	Ac-FWY-OH		
7	TSC**1**-FFY-OH	–	77% (250 μM)
8	TSC**1**-FYY-OH	6.57 ± 0.5	–
9	TSC**1**-FWY-OH	6.66 ± 0.5	–
10	TSC**2**-FFY-OH	–	45% (250 μM)
11	TSC**2**-FYY-OH	–	36% (250 μM)
12	TSC**2**-FWY-OH	–	50% (250 μM)
13	TSC**3**-FFY-OH	–	62% (250 μM)
14	TSC**3**-FYY-OH	26.12 ± 9	–
15	TSC**3**-FWY-OH	25.14 ± 2.5	–

### Melanogenesis inhibition

The effect of the synthetic compounds on melanogenesis inhibition was performed in B16F0 murine melanoma cell line. The mouse model of human skin is often employed in skin biology and cancer research due to numerous similarities.^62^ B16F0 murine melanoma cell line shares most of the melanogenesis with human melanocytes. Therefore, it is the proper model for screening the effect of potential inhibitors on melanogenesis.^63^

The results of percentage inhibition of melanogenesis by synthetic compounds **1–15** as well as dose-response measurements (IC_50_) along with confidence intervals for melanin production and cell proliferation in B16F0 mouse melanoma cells are shown in Table 2. Kojic acid was used as a standard control, DMSO was used as a solvent control, unconjugated thiosemicarbazone TSC **1–3** and tripeptide sequences **4–6** were used as controls for TSC-tripeptide conjugates **7–15**.

**Table 2.** Results of inhibition of melanin production and cell proliferation in B16F0 cell line.

Compound				Obtained values (*n* = 3)			
		% Of melanogenesis inhibition at a specific inhibitor concentration		IC_50_^a^ of melanin production [µM]	95% CI^b^ IC_50_ [µM]	IC_50_^a^ of cell proliferation [µM]	95% CI^b^ IC_50_ [µM]
		40 µM	10 µM				
Kojic acid		12	1	121.0	105.5–138.8	162.2	116.6–225.6
DMSO		4	2	n/a^c^	n/a	144.6	114.9–181.9
1	TSC**1**	30	14	101.5	88.38–116.6	113.6	90.50 − 142.6
2	TSC**2**	77	28	19.8	17.3–22.6	71.5	48.4 – 105–6
3	TSC**3**	13	1	126.4	109.3–146.2	113.1	101.4 − 126.3
4	Ac-FFY-OH	3	1	245.7	58.5–1033	106.2	76.94–146.6
5	Ac-FYY-OH	2	0	174.7	79.5–383.8	96.6	80.07 − 116.5
6	Ac-FWY-OH	4	0	140.9	104.5–189.9	81.0	62.74 − 104.6
7	TSC**1**-FFY-OH	10	1	138.4	114.1–167.9	72.8	62.95 − 84.23
8	TSC**1**-FYY-OH	12	3	158.5	131.8–190.6	99.0	82.07 − 119.5
9	TSC**1**-FWY-OH	11	0	150.6	123.5–183.7	77.1	59.49 − 99.96
10	TSC**2**-FFY-OH	4	0	146.1	99.0–215–5	77.4	55.52–107.9
11	TSC**2**-FYY-OH	0	0	n/a	n/a	77.2	68.76 − 86.38
12	TSC**2**-FWY-OH	3	0	89.9	74.87–107.9	63.2	49.67–80.38
13	TSC**3**-FFY-OH	3	0	162.4	113.0–233.5	49.8	35.55 − 69.89
14	TSC**3**-FYY-OH	4	0	144.9	110.1–190.8	83.7	68.94 − 101.6
15	TSC**3**-FWY-OH	20	5	67.1	63.8–70.7	67.5	59.58 − 76.52

^a^Inhibitor concentration, which is required for 50% inhibition.

^b^Confidence interval.

^c^Not applicable.

The results show that melanogenesis in B16F0 cells was inhibited by all the synthetic compounds **1–15** (except for compound **11**), kojic acid, and DMSO in a dose-dependent manner. Almost all compounds (**3–14**) inhibited 0–3% of melanogenesis at the concentration of 10 µM, and 4–13% at 40 µM, which does not place them among the most potent melanogenesis inhibitors. Only TSC **1–3** and the tripeptide conjugate **15** inhibited melanogenesis more than kojic acid at these two concentrations.

Interestingly, TSC **2** possessing the bulkiest structure turned out to be the most potent melanogenesis inhibitor. It inhibited 77% of melanogenesis activity at 40 µM. This result is in agreement with the results of one of our previous papers^48^ where thiosemicarbazone of benzophenone and of (*E*)-2-phenylacetophenone (possessing an additional aromatic bulky substituent) revealed IC_50_ value for melanogenesis below 2 µM. Interestingly, this phenomenon seemed to be independent of the tyrosinase inhibition, as thiosemicarbazone of benzophenone was an efficient tyrosinase inhibitor, while thiosemicarbazone of (*E*)-2-phenylacetophenone did not inhibit tyrosinase at all.

**Figure 3.**
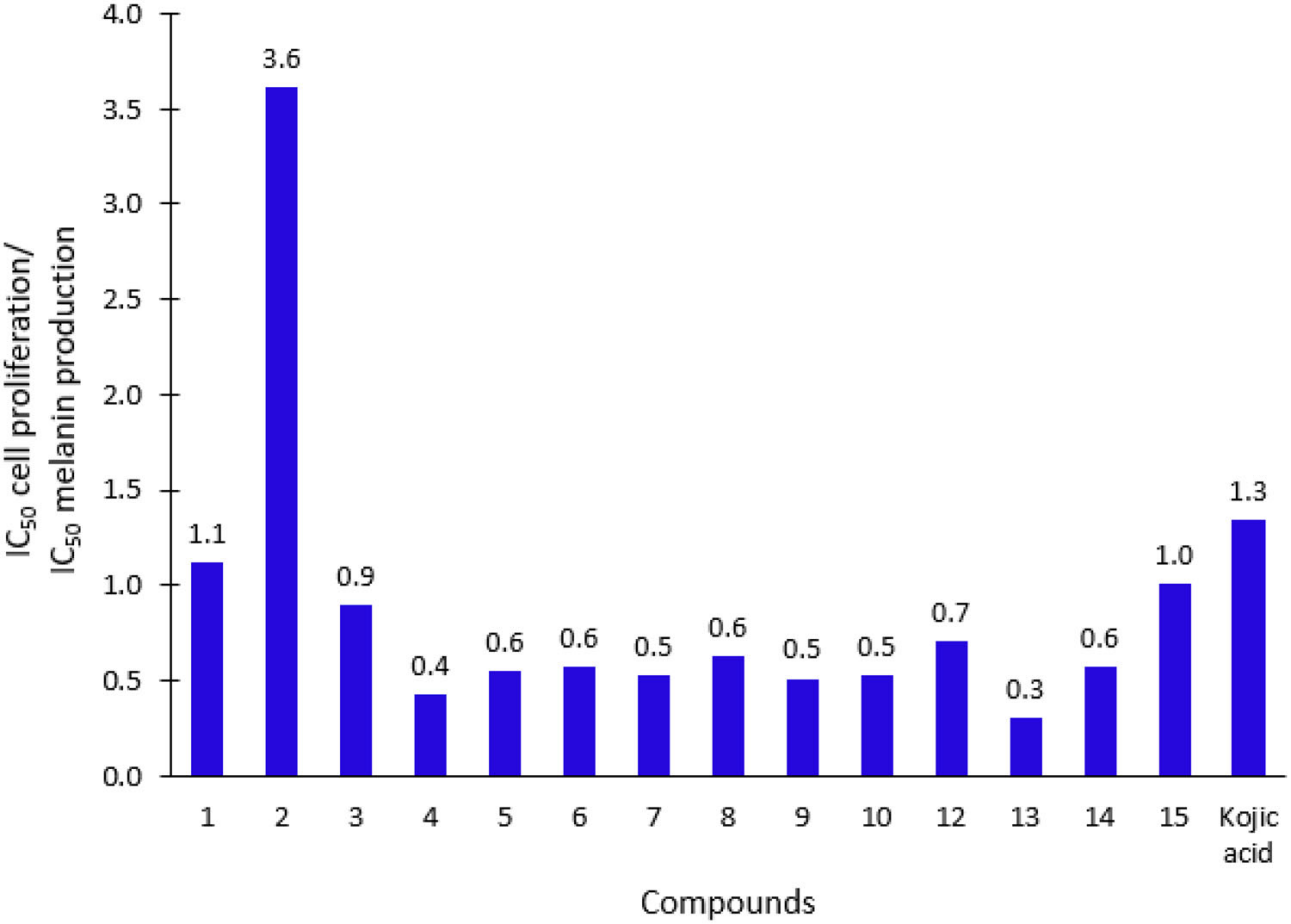
Inhibition of B16F0 cell proliferation *versus* inhibition of melanin production. The bar graph represents the ratio of IC_50_ for B16F0 cell proliferation to IC_50_ referred to melanin production, counted for all investigated compounds (except compound **11**) and kojic acid.

All the investigated compounds (except for compound **11**) revealed IC_50_ at the micromolar level. TSC **1** and **2** as well as the tripeptide conjugates **12** and **15** showed stronger potency of melanogenesis inhibition than the positive control kojic acid (lower IC_50_ values in comparison with kojic acid, which gave IC_50_ of 121 µM). These results were verified with the effect of tested inhibitors on the proliferation of B16F0 cells (Table 2). Low melanin production might have been caused by high toxicity (low IC_50_ of inhibition of cell proliferation) of the inhibitors which, in turn, can lead to inhibition of melanin production. IC_50_ of cell proliferation is in the range of 49.8–113.6 µM, and all are lower than kojic acid (162.2 µM). The investigated compounds are not highly toxic to B16F0 cells but at the same time, they inhibit melanogenesis with relatively low efficacy. The ratio between the potency of TSCs to inhibit melanin production *vs.* cell toxicity is visualised in Figure 3.

The higher bars in Figure 3 indicate the higher ratio of melanogenesis inhibition to toxicity. Low ratio values, especially <1.0, do not let to conclude if melanin production inhibition depends on the direct activity of the inhibitor or on the inhibition of cell proliferation. Therefore, IC_50_ of melanin production should be higher than IC_50_ of cell proliferation. The only suitable ratio refers to TSC **2** (ratio: 3.6) and this is the only thiosemicarbazone displaying a better ratio value than kojic acid (ratio: 1.3). TSC **2** inhibits melanogenesis in B16F0 cell line but shows no significant inhibition towards tyrosinase. It appears that TSC **2** inhibits the pathway at different stages than the step catalysed by tyrosinase, for example affecting the activity of other key enzymes in melanin biosynthesis, e.g. dopachrome tautomerase, microphthalmia-associated transcription factor, or tyrosinase c protein 1.^64^

### Molecular docking

Understanding the nature ofguest–host interactions is mandatory for the rational design of inhibitors. Molecular modelling, especially simulation of the docking process, can contribute to understanding the physical nature of those interactions. Performing docking simulations allow us to better understand the results of biological tests. During the preliminary steps, structures of the investigated thiosemicarbazones and their conjugates with the designed tripeptide sequences have been optimised by using *Gaussian16* code at the B3LYP/6-311g(d,p) level with a solvent model and water as solvents. In the case of TSC **2**, containing an asymmetric carbon atom, both R and S isomers have been considered. Isomer *E* was docked as the default configuration, however during the docking process there was a possibility to freely change between *E* and *Z*. No significant results were found for *Z* isomer; therefore, all the presented data refer to the preferential *E* configuration. In the second preliminary step, the structure of tyrosinase was protonated at pH 6.8 using *H++* web server^54^^,^.^55^ Tyrosinase structure has been obtained from Protein Data Bank (PDB ID: 2Y9X).^53^ Calculated structures of inhibitors were docked to the active site of tyrosinase by using *Gold ver. 2021.3* algorithm.^65^ Ten orientations of each investigated inhibitor were obtained, among which the ones with the best value of the ChemPLP scoring function were selected for further investigations.

As it was shown in our previous results of docking studies, the thiourea moiety of thiosemicarbazone inhibitors usually interacts with copper ions of tyrosinase molecule.^46–48^ Because of that, according to the previously published procedure, we adopted a docking protocol without any additional inhibitor-enzyme constraints.

**Figure 4.**
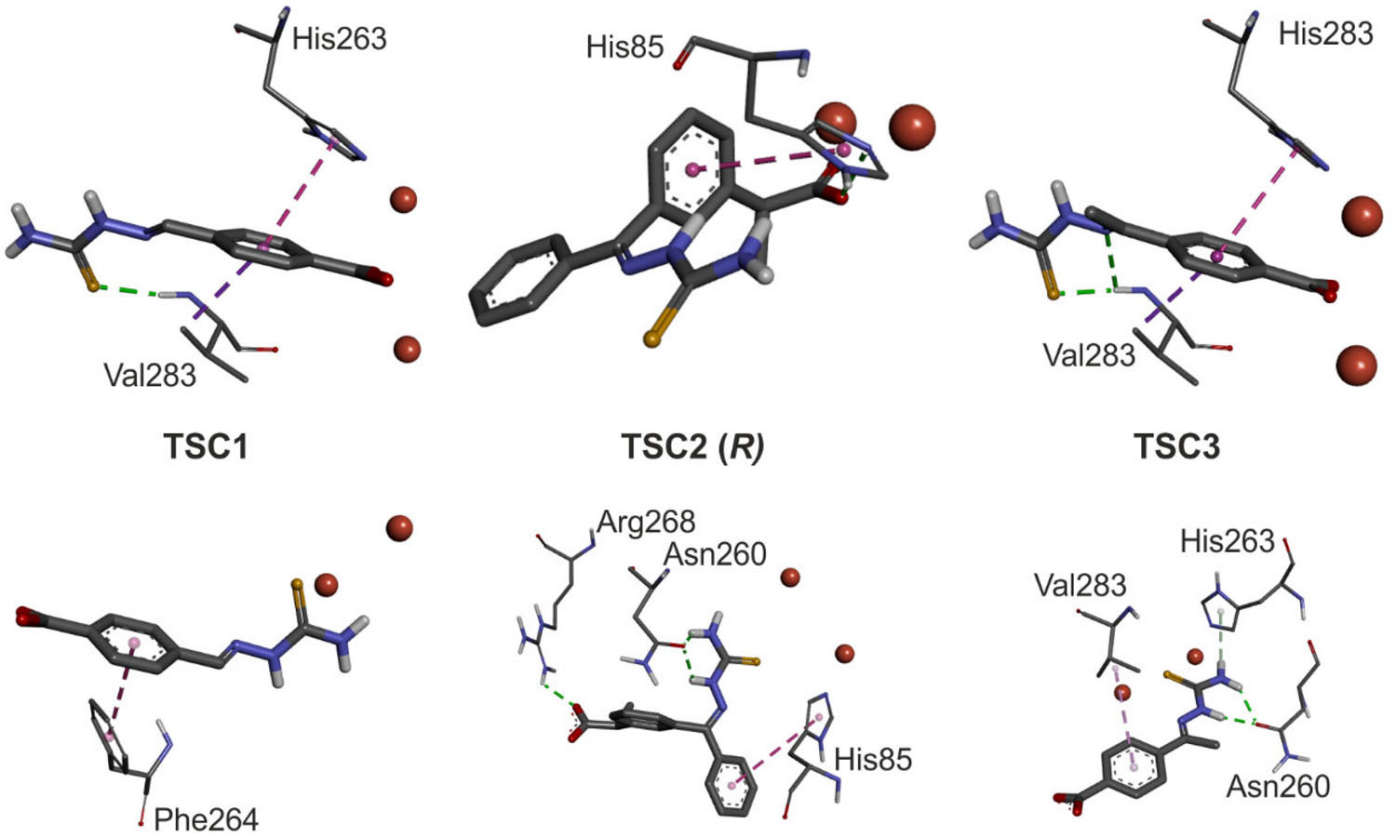
Orientation of the thiosemicarbazone TSC **1**–**3** in the tyrosinase active site. Top: free docking; bottom: constrained docking. Intermolecular interactions are represented as dash lines – green: H-bond; purple: π-π interaction. Copper ions are shown as red spheres.

Structural analysis of inhibitor-enzyme complexes for TSC **1**–**3** shows that the alignment of each thiosemicarbazone inhibitor is stabilised by π-π stacking interaction between the inhibitor phenyl moiety and the imidazole ring of His263 (see Figure 4, top). Additionally, strong interaction between the negatively charged carboxylate moiety of inhibitors and copper ions (see **Figure SI 32**) firmly anchors them into the active site.

These unexpected results deriving from calculations show that inhibitors TSC **1**–**3** interact with the enzyme not through the thiourea moiety, but through the carboxylate one. To confirm this unexpected preference, we performed docking studies using additional structural constraints, forcing the distance between the sulphur atom of the thiourea group and copper ions in the range of 1.5–5.0Ang. The use of these structural constraints allowed us to obtain distances in which the thiourea group interacted with copper ions (see Figure 4 bottom). However, comparing the scoring function values we observed that these positions are less preferred regardless of the considered inhibitor (see **Table SI 2**). Similar conclusions can be drawn from comparing free and constrained docking results for peptide conjugates. Nevertheless, in the case of these inhibitors, differences are not significant.

The relationship between inhibitory activity and the sequence of the tripeptide linked in the conjugates can be analysed using the example of thiosemicarbazone TSC **1**. As mentioned above, the scores of the scoring function obtained in free and constrained docking are relatively similar, suggesting that interaction *via* the thiourea group is also possible. Therefore, we analysed the impact of the tripeptide sequence on the ability to interact with the protein surface for both docking variants.

Generally, in the case of free docking, the small cavity binding of both copper ions is occupied mainly by the phenyl moiety of Phe1 side-chain of the tripeptide conjugate. Similar behaviour is also found for the other thiosemicarbazone conjugates. Only a few distances, less than 5% of all obtained orientations, present different ways of impact. Apart from the minimal frequency of their occurrence, they are also characterised by lower values of the scoring function. This observation is a bit surprising in the face of the results summarised in,^22^ where interaction through the tyrosine side-chain, which is a natural substrate, is suggested.

**Figure 5.**
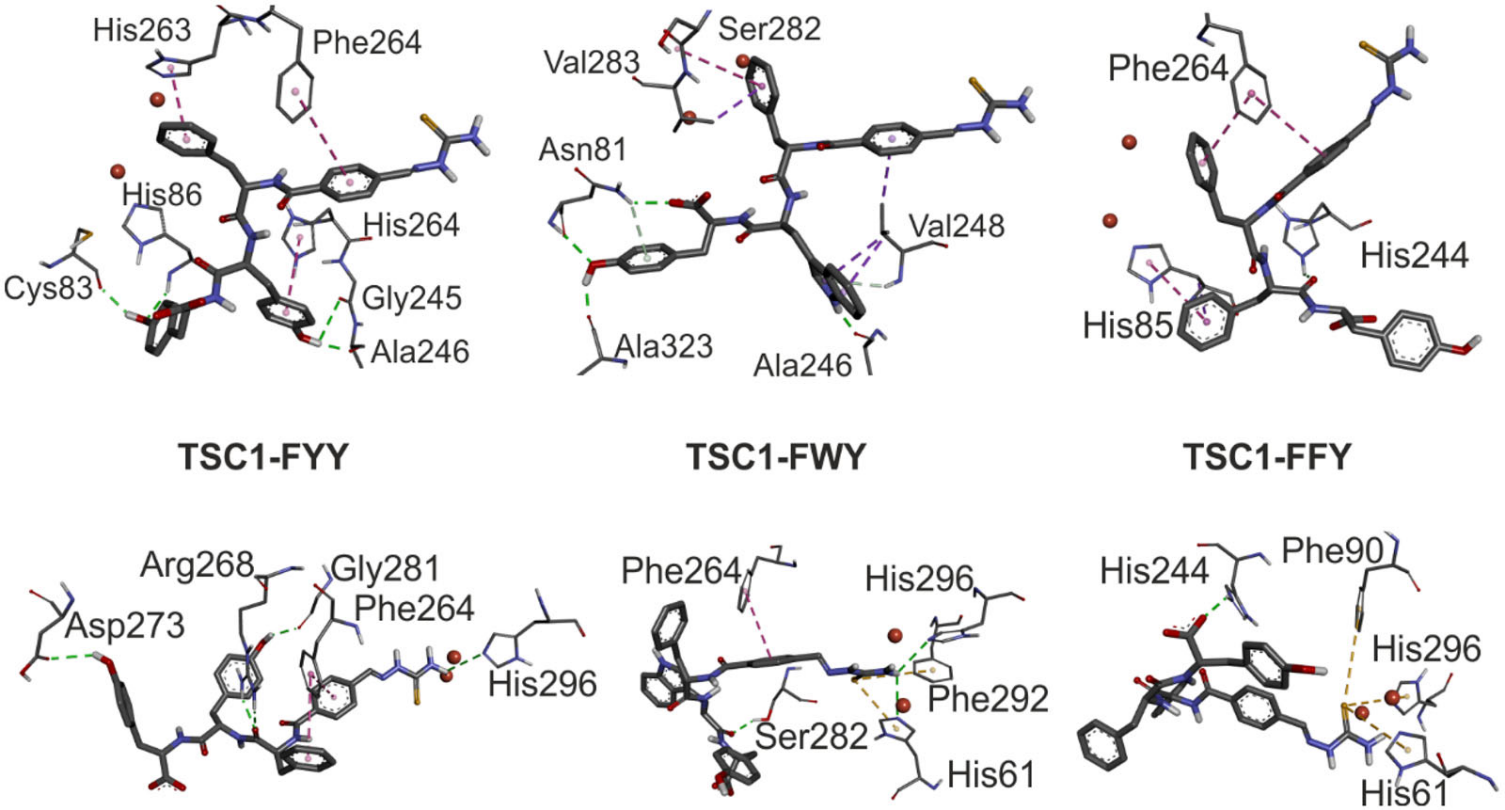
Orientation of the thiosemicarbazone TSC **1** tripeptide conjugates in the tyrosinase active site. Top: free docking; bottom: constrained docking. Intermolecular interactions are shown as dash lines – green: H-bond; purple: π-π interaction. Copper ions are shown as red spheres.

Comparing the arrangement of the tripeptide conjugates TSC**1**-FYY (**8**), TSC**1**-FWY (**9**), and TSC**1**-FFY (**7**), the main differences can be found in how the peptide side-chains interact with the tyrosinase surface. For the TSC**1**-FYY (**8**) and TSC**1**-FWY (**9**) inhibitors, the highest number of intermolecular hydrogen bonds has been found. The position of TSC**1**-FYY (**8**) in the enzymatic active site is stabilised by four intermolecular hydrogen bonds involving hydroxyl groups of Tyr2 and Tyr3 sidechains. The alignment of TSC**1**-FWY (**9**) is stabilised by three intermolecular hydrogen bonds, involving indole amide proton of Trp2 and hydroxyl group of Tyr3 of the inhibitor. The orientation of the TSC**1**-FFY (**7**) in the tyrosinase active site is stabilised only by two intermolecular hydrogen bonds, involving carbonyl oxygen (in the backbone) from Phe2 and Tyr3. Detailed information on the formed hydrogen bonds is presented in Table 3. Moreover, the alignments of all inhibitors are stabilised by π-π interactions (TSC**1**-FYY (**8**): π-π stacking Phe1-His263; π-π edge-to-face Ph(TSC**1**)-Phe264; Tyr2-His244; TSC**1**-FFY (**7**): π-π edge-to-face Ph(TSC**1**)-Phe264). Additionally, in the case of TSC**1**-FYY (**8**) and TSC**1**-FWY (**9**) some stabilising interactions of the sulphur atom of the thiourea group with the π-electrons of the Trp227 indole ring can also be observed. Obtained orientations of the investigated inhibitors in the active site are shown in Figure 5 (top). In contrast, in the case of constrained docking, the alignment of each inhibitor is stabilised by the π-electron-sulphur atom interaction. This result seems to be expected due to the fact that such an orientation of the inhibitors towards the active cavity is forced.

**Table 3.** List of the residues of inhibitors **7–9** and tyrosinase, involved in the intermolecular hydrogen bonds formed during the free and constrained docking.

Inhibitor				Tyrosinase	
Compound		Residue	Functional group	Residue	Functional group
**Free docking**	TSC**1**-FYY (**8**)	Tyr2	OH	Gly245	CO
		Tyr2	OH	Ala246	CO
		Tyr3	OH	Cys83	CO
		Tyr3	OH	His85	HN^amide^
	TSC**1**-FWY (**9**)	Trp2	HN^indol^	Ala246	CO
		Tyr3	OH	Ala323	CO
		Tyr3	OH	Asn81	HN
	TSC**1**-FFY (**7**)	Phe2	CO^a^	His244	HN^imidazole^
		Tyr3	CO_2_^a^	Val248	HN^amide^
**Constrained docking**	TSC**1**-FYY (**8**)	TSC **1**	H_2_N	His296	N^imidazole^
		Phe1^b^	CO	Arg268	H_2_N^guanidine^
		Tyr2	HO	Gly281	CO
		Tyr3	HO	Asp273	CO_2_^sidechain^
	TSC**1**-FWY (**9**)	TSC **1**	H_2_N	His61	N^imidazole^
		TSC **1**	H_2_N	His296	N^imidazole^
		Trp2	CO^a^	Ser282	HO^sidechain^
	TSC**1**-FFY (**7**)	Tyr3	CO_2_^a^	His244	HN^imidazole^

^a^Peptide backbone.

^b^Double interaction.

The sulphur atom of thiourea moiety interacts mainly with the aromatic ring of His61, Phe90, and His296, regardless of the peptide sequence of the conjugates. As in the case of free docking and constraint docking, differences in the strength of the interaction of inhibitors with the enzyme can be explained on the basis of the number of intermolecular hydrogen bonds. Similarly, the arrangement of TSC**1**-FYY (**8**) is stabilised by the highest number of hydrogen bonds (Table 3). Moreover, in the case of TSC**1**-FWY (**9**) the observed interaction formed by Trp2 involves the tryptophane backbone instead of the amino acid side chain. The orientations obtained for the investigated inhibitors in the active site are shown in Figure 5 (bottom).

The presented results allowed us to conclude that introducing an amino acid in the second position without the donor hydrogen bond in the side chain causes a drastic decrease in the ability to form intermolecular hydrogen bonds. This, in turn, significantly limits the inhibitory properties, which is reflected in the values of the scoring function. The inhibitory properties of the tripeptide conjugates containing tyrosine or tryptophan in the second position are comparable. On the other hand, inhibitors containing a phenylalanine residue at this position have a much lower affinity for the tyrosinase active cavity.

## Conclusions

Conjugation of peptides with small organic molecules has been recently widely applied, mainly for their application as carriers or stabilising agents.^25–27^ An interesting example is represented by the use of various peptides and their conjugates as cosmeceutical active ingredients, described in recent reviews^15^^,^.^16^ In this work, we report the synthesis of nine conjugates of three thiosemicarbazones with designed tripeptides, based on the Phe-Xaa-Tyr motif, where Xaa = Phe, Trp, or Tyr. Our aim was to evaluate the tyrosinase inhibitory potential of such conjugates, following the encouraging results published in the past years when we demonstrated the strong inhibitory properties of various thiosemicarbazones. Furthermore, several examples of peptide conjugates, e.g. with a commercially available inhibitor of tyrosinase, such as kojic acid, resulted in increased inhibitory activity and/or enhanced stability, or decreased cytotoxicity of the final product.

New thiosemicarbazone structures (TSCs) were selected considering our previous results, demonstrating that the thiourea group is responsible for copper coordination in the tyrosinase active cleft. Considering these data, we designed three new thiosemicarbazones with a free carboxyl group, located distantly from the active thiourea moiety. Therefore, three new TSCs possessing free carboxyl moieties necessary for the conjugation were synthesised. Tripeptide sequences were selected on the base of previously published data, suggesting the relevance of N-terminally located Phe residue and the fundamental role of aromatic and hydrophobic residues for tyrosinase inhibition. TSCs were conjugated to the tripeptide chains in the solid phase *via* standard coupling strategy, by N-terminal modification *via* amide bond formation. Final conjugates were cleaved from the resin, purified, and characterised. Pure products were then evaluated as inhibitors of tyrosinase, demonstrating a good inhibitory activity of two structurally similar thiosemicarbazones TSC **1** and **3**, and poor activity of the bulky TSC **2** (IC_50_ 5.3 and 5.8 μM *versus* > 150 μM, respectively). At variance, the unmodified peptides **4**–**6** did not display significant inhibition, in accordance with the results from the literature (e.g. IC_50_ of Ac-FFY-OH found in the literature: 240 μM).^23^ Finally, we tested the conjugates, observing that the tripeptide conjugates containing TSC **2** showed weak inhibitory activity, the set of TSC **1**-derivatives **7**–**9** contains the most active compounds **8** and **9** (IC_50_ value: 6.5 μM for both conjugates), while the conjugates of TSC **3**, compounds **14** and **15**, feature similar intermediate activity (IC_50_ around 25 μM). Surprisingly, we found that the derivatives including FFY sequence (compounds **7** and **13**) are significantly weaker inhibitors of tyrosinase regarding FWY and FYY conjugates (IC_50_ >150 μM). Molecular modelling supports the explanation of the influence of the amino acid sequence on the activity of conjugates. *In silico* results showed that the reason for significant differences in the activity of Phe-containing derivatives should be sought in the lack of a hydrogen bond donor group, caused by introducing a phenylalanine residue in the central position of the peptide.

Tests performed on B16F0 cell line show that all investigated compounds inhibited melanogenesis at a micromolar level. However, only TSC **1** and **2** as well as the tripeptide conjugates **12** and **15** showed stronger potency of melanogenesis inhibition than kojic acid, so they can be considered as medium melanogenesis inhibitors. It should be noted that bulky thiosemicarbazone TSC **2**, unexpectedly, revealed the highest inhibitory potency towards melanogenesis showing relatively low toxicity towards the cells. TSC **2** showed no significant inhibition towards tyrosinase, so it probably inhibits melanogenesis at a different stage of the process.

For most compounds, IC50 of cell proliferation inhibition turned out to be lower than IC_50_ of melanin production. This observation lets raise the question if the inhibited melanin production is the result of inhibitor activity towards melanogenesis process, or of toxic effect on the cells. Further investigations to explain the mechanism of inhibition of tyrosinase in normal cells, and the role of peptide sequence in this mechanism should be performed in further studies.

According to the results of molecular modelling, in the case of inhibitors **1–3**, their interaction with the active cavity of the enzyme through free carboxyl groups should be taken into account. This interaction seems so significant that it may force an orientation opposite to that postulated for this class of compounds.^48^ Our previous studies indicated that introducing an additional methyl group at the *R* position significantly improved inhibitory activity. This could be explained by a better fit for the active cave and, thus, an increase in van der Waals interactions, which would only occur thanks to the interaction *via* the thiourea moiety. On the other hand, similar tyrosinase inhibiting abilities of thiosemicarbazones **1** and **3** presented in this work indicate that the presence of this additional methyl group does not have such a significant effect on the activity, which would suggest their different interaction with the enzyme. Moreover, the differences in the number of interactions between TSC **1** and derivatives TSC**1**-FYY (**8**) and TSC**1**-FWY (**9**) are significant. While TSC **1** interacts with tyrosinase through π-π stacking interaction provided by the TSC **1** phenyl moiety, peptide derivatives **8** and **9** form numerous hydrogen bonds between amino acid residues and the tyrosinase. Peptide conjugates **8** and **9** maintain the *in vitro* inhibitory properties of TSC **1** alone, but it should be noticed that the interactions observed *in silico* of the peptide chain and tyrosinase active cleft significantly stabilise the complex.

The above-mentioned results might be helpful in designing and developing novel skin-whitening products based on tyrosinase inhibitors.

## Supplementary Material

Supplemental Material
