# Supplementary material for: Tripeptides conjugated with thiosemicarbazones: new inhibitors of tyrosinase for cosmeceutical use
Source: J Enzyme Inhib Med Chem. 2023 May 5;38(1):2193676. doi: 10.1080/14756366.2023.2193676 (PMC10165932; doi:10.1080/14756366.2023.2193676)
Supplement: Supplemental Material [file IENZ_A_2193676_SM5991.pdf]

Supplementary Information (SI)

for the manuscript

Tripeptides conjugated with thiosemicarbazones: new inhibitors of tyrosinase for cosmeceutical use

Patrycja Ledwoń[a,b], Waldemar Goldeman[c], Katarzyna Hałdys[a], Michał Jewgiński[a], Greta Calamai[b], Joanna Rossowska[d], Anna Maria Papini[e], Paolo Rovero[b*], and Rafał Latajka[a*]

[a] *Department of Bioorganic Chemistry, Faculty of Chemistry, Wroclaw University of Science and Technology, Wybrzeże Stanisława Wyspiańskiego 29, 50-370 Wrocław, Poland*

[b] *Interdepartmental Research Unit of Peptide and Protein Chemistry and Biology, Department of Neurosciences, Psychology, Drug Research and Child Health Section of Pharmaceutical Sciences and Nutraceutics, University of Florence, Via Ugo Schiff 6, 50019 Sesto Fiorentino, Firenze, Italy*

[c] *Department of Organic and Medicinal Chemistry, Faculty of Chemistry, Wroclaw University of Science and Technology, Wybrzeże Stanisława Wyspiańskiego 29, 50-370 Wrocław, Poland*

[d] *Ludwik Hirszfeld Institute of Immunology and Experimental Therapy, Polish Academy of Science, ul. Rudolfa Weigla 12, 53-114 Wrocław, Poland*

[e] *Interdepartmental Research Unit of Peptide and Protein Chemistry and Biology, Department of Chemistry "Ugo Schiff", University of Florence, Via della Lastruccia 13, 50019 Sesto Fiorentino, Firenze, Italy*

***** Correspondence: paolo.rovero@unifi.it (PR), rafal.latajka@pwr.edu.pl (RL)

Information Classification: General

LIST OF TABLES

Table SI 1. Analytical data for compounds **4-15**. Gradient applied: 30-70% B in A in 5'. 5
Table SI 2. Comparison of ChemPLP scoring values for free and constrained docking protocols. 29

LIST OF FIGURES

Figure SI 1. ^{1}H NMR spectrum of TSC **1**. 6
Figure SI 2. Expanded ^{1}H NMR spectrum of TSC **1**. 6
Figure SI 3. ^{13}C{^{1}H} NMR spectrum of TSC **1**. 7
Figure SI 4. Expanded ^{13}C{^{1}H} NMR spectrum of TSC **1**. 7
Figure SI 5. ^{1}H NMR spectrum of TSC **2**. 8
Figure SI 6. Expanded ^{1}H NMR spectrum of TSC **2**. 8
Figure SI 7. Expanded ^{1}H NMR spectrum of TSC **2**. 9
Figure SI 8. ^{13}C{^{1}H} NMR spectrum of TSC **2**. 9
Figure SI 9. Expanded ^{13}C{^{1}H} NMR spectrum of TSC **2**. 10
Figure SI 10. Expanded ^{13}C{^{1}H} NMR spectrum of TSC **2**. 10
Figure SI 11. Expanded ^{13}C{^{1}H} NMR spectrum of TSC **2**. 11
Figure SI 12. ^{1}H NMR spectrum of TSC **3**. 12
Figure SI 13. Expanded ^{1}H NMR spectrum of TSC **3**. 12
Figure SI 14. ^{13}C{^{1}H} NMR spectrum of TSC **3**. 13
Figure SI 15. Expanded ^{13}C{^{1}H} NMR spectrum of TSC **3**. 13
Figure SI 16. Chromatogram of **4** (Ac-FFY-OH) and MS spectra of the peak 2.7 min. 14
Figure SI 17. Chromatogram of **5** (Ac-FYY-OH) and MS spectra of the peak 1.4 min. 15
Figure SI 18. Chromatogram of **6** (Ac-FWY-OH) and MS spectra of the peak 2.5 min. 16
Figure SI 19. Chromatogram of **7** and MS spectra of the peak 3.4 min. 17
Figure SI 20. Chromatogram of **8** and MS spectra of the peak 3.8 min. 18
Figure SI 21. Chromatogram of **9** and MS spectra of the peak 3.5 min. 19
Figure SI 22. Chromatogram of **10** and MS spectra of the peaks found. 20
Figure SI 23. Chromatogram of **11** and MS spectra of the peaks found. 21
Figure SI 24. Chromatogram of **12** and MS spectra of the peaks found. 22
Figure SI 25. Chromatogram of **13** and MS spectra of the peaks found. 23
Figure SI 26. Chromatogram of **14** and MS spectra of the peaks found. 24
Figure SI 27. Chromatogram of **15** and MS spectra of the peaks found. 26
Figure SI 28. Percentage of tyrosinase activity in the presence of compounds **1-3**. 27
Figure SI 29. Percentage of tyrosinase activity in the presence of compounds **7-9**. 27
Figure SI 30. Percentage of tyrosinase activity in the presence of compounds **10-12**. 28
Figure SI 31. Percentage of tyrosinase activity in the presence of compounds **13-15**. 28
Figure SI 32. Distance values between the carboxylic group of inhibitor and enzyme's copper ions. (TSC **1** - left, TSC **3** - right). Copper ions are shown as brown spheres. 29

Information Classification: General

1. *Synthesis of thiosemicarbazones*

Preparation of TSC 1 and TSC 3. A mixture of 4-acetylbenzoic acid (5.0 mmol, 821 mg) or 4-formylbenzoic acid (5.0 mmol, 751 mg) and thiosemicarbazide (5.0 mmol, 456 mg) in 96% ethanol (25 mL) was refluxed for about 12 hours (in both case the precipitate starts to form after 15-30 min of heating). After cooling, the reaction mixture was left for 24 h at room temperature. The resulting solid was filtered, washed with water (5 × 5 mL), 96% ethanol (4 × 5 mL), diethyl ether (2 × 20 mL) and dried on air giving TSC **1** and TSC **3** as white fine crystalline solids.

(*E*)-4-Formylbenzoic acid thiosemicarbazone (TSC **1**): Yield 91 %; ^{1}H NMR (400MHz, DMSO-d$_6$) δ: 7.86-7.91 (m, (AA'BB' spin system), 4H, Ar*H*), 8.05 (s, 1H, C*H*=N), 8.07 (bs, 1H, N*H*), 8.26 (bs, 1H, N*H*), 11.53 (s, 1H, N*H*), 13.00 (bs, 1H, COOH); ^{13}C {^{1}H} NMR (100 MHz, DMSO-d$_6$) δ: 127.59, 130.06, 132.02, 138.34, 141.49, 167.64, 178.94; HRMS (ESI+): *m/z* calculated for $C_9H_{10}N_3O_2S$ $(M+H)^+$ 224.0494, found 224.0495. ^{1}H NMR spectrum is in agreement with data reported in the literature[1,2].

(*E*)-4-Acetylbenzoic acid thiosemicarbazone (TSC **3**): Yield 85 %; ^{1}H NMR (400MHz, DMSO-d$_6$) δ: 2.28 (s, 3H, CH$_3$), 7.87 (d, 2H, *J*=8.4 Hz, Ar*H*), 8.00 (bs, 1H, N*H*), 8.01 (d, 2H, *J*=8.4 Hz, Ar*H*), 8.33 (bs, 1H, N*H*), 10.30 (s, 1H, N*H*), 12.98 (bs, 1H, COOH); ^{13}C {^{1}H} NMR (100 MHz, DMSO-d$_6$) δ: 19.24, 131.94, 134.44, 136.19, 146.94, 151.89, 172.35, 184.39; HRMS (ESI+): *m/z* calculated for $C_{10}H_{12}N_3O_2S$ $(M+H)^+$ 238.0650, found 238.0654.

Preparation of TSC 2. A mixture of racemic ketoprofen (2.0 mmol, 509 mg), thiosemicarbazide (6.0 mmol, 547 mg), and *p*-toluenesulfonic acid monohydrate (2.0 mmol, 380 mg) in 2-propanol (25 mL) was refluxed for about 12 h. After cooling, the reaction mixture was left for 24 h at room temperature. The resulting solid of excess of thiosemicarbazide was filtered, washed with 2-propanol (5 × 3 mL) and discarded. The filtrate was evaporated to dryness. The semisolid residue was treated with DCM (10 mL) and 5% $NaHCO_3$ aqueous solution (25 mL) and the two-phase mixture was stirred for about 30 min at room temperature. The organic phase was separated, the aqueous phase was extracted with DCM (5×2 mL) and the combined organic phases were discarded. Then the aqueous phase was carefully neutralized with 6M HCl and extracted with DCM (3×10 mL). The combined organic phases were dried over anhydrous Na_2SO_4 and evaporated to dryness giving TSC **2** as a thick oil that crystallized during *in vacuo* drying to give finally a light beige solid.

Mixture of rac-(*E*)-ketoprofen thiosemicarbazone and rac-(*Z*)-ketoprofen thiosemicarbazone (TSC **2**): Yield 51%; ^{1}H NMR (400MHz, DMSO-d$_6$) δ: 1.30 (d, *J*=7.2 Hz, CH$_3$) and 1.37 (d, *J*=7.2 Hz, CH$_3$) [total integration 3H], 3.65 (q, *J*=7.2 Hz, CH) and 3.77 (q, *J*=7.2 Hz, CH) [total integration 1H], 7.15-7.40 (m, 5H, Ar*H*), 7.47-7.70 (m, 4H, Ar*H*), 8.33 (bs, N*H*) and 8.38 (bs, N*H*) [total integration 2H], 8.60 (bs, N*H*) and 8.64 (s, N*H*) [total integration 1H], 12.35 (bs, 1H, COOH); ^{13}C {^{1}H} NMR (100 MHz, DMSO-d$_6$) δ: 19.06 and 19.11 (CH$_3$), 44.99 and 45.11 (CH), 126.95, 127.17, 127.21, 127.91, 128.10, 128.80, 128.90, 129.02, 129.14, 129.52, 130.31, 130.38, 130.49, 130.55, 131.70, 131.78, 136.81 and 136.91, 142.12 and 143.55, 149.25, 149.55, 175.56, 175.70, 178.31, 178.38; HRMS (ESI+): *m/z* calculated for $C_{17}H_{18}N_3O_2S$ $(M+H)^+$ 328.1120, found 328.1117. Ketoprofen thiosemicarbazone is described in the literature, however with incomplete NMR data[3] or without any spectroscopic data[4].

Information Classification: General

2. *Solid-phase peptide synthesis*

Before the synthesis, dry Fmoc-Tyr(*t*Bu)-Wang resin was swelled for 40 min in DMF. Fmoc-deprotection was performed with a solution of 20% (v/v) piperidine in DMF. Peptide chain elongation was performed by repeated cycles of deprotection, washings, and coupling with Fmoc-protected amino acids or TSCs (2.5 eq.), HBTU (2.5 eq.), and DIPEA (4.5 eq.) in DMF. Coupling reactions were performed for 40 min, deprotection reactions for 5+15 min, and washings with DMF between each step for 4×1 min. Uncertain couplings were confirmed by the ninhydrin test described by Kaiser[5]. Unmodified sequences were acetylated N-terminally before the cleavage, using a solution of Ac_2O (20 eq.) and NMM (20 eq.) in DCM for 2×45 min. Final cleavage and side-chain deprotections were performed using a mixture of TFA/TIS/H_2O (95/2.5/2.5, v/v/v) at room temperature. The solution was filtered off, resin was washed additionally with 1 mL of the cleavage cocktail, and TFA was evaporated under N_2 flux. Then, cold diethyl ether was added for peptide precipitation. Precipitated peptides and their conjugates were centrifuged and washed additionally with Et_2O (2×), then lyophilized.

1. Liu K, Lu H, Hou L, Qi Z, Teixeira C, Barbault F, Fan B-T, Liu S, Jiang S, Xie L. 2008. Design, Synthesis, and Biological Evaluation of *N* -Carboxyphenylpyrrole Derivatives as Potent HIV Fusion Inhibitors Targeting gp41. J Med Chem. 51(24):7843–7854. doi:10.1021/jm800869t

2. Z. Laczkowski K, Biernasiuk A, Baranowska-Laczkowska A, Misiura K, Malm A, Plech T, Paneth A. 2016. Synthesis, Antibacterial Activity, Interaction with Nucleobase and Molecular Docking Studies of 4-Formylbenzoic Acid Based Thiazoles. MC. 12(6):553–562. doi:10.2174/1573406412666160201121310

3. Allawi MM, Mahdi M, Raoof A. 2021. Synthesis, preliminary pharmacological evaluation, molecular docking, and ADME studies of new 4-thiazolidinone derivatives bearing ketoprofen moiety targeting cyclooxygenase enzyme. Egypt J Chem. 64(12):7339–7350. doi:10.21608/ejchem.2021.71012.3561

4. Saha DK, Padhye S, Padhye S. 2001. Targeting Estrogen Receptor Sites in Human Breast Cancer Cell Line T47D With Copper Conjugates of Nonsteriodal Anti-inflammatory Drug Derivatives: Antiproliferative Activity of Ketoprofen Derivative and its Copper Complex. Metal-Based Drugs. 8(2):73–77. doi:10.1155/MBD.2001.73

5. Kaiser E, Colescott RL, Bossinger CD, Cook PI. 1970. Color test for detection of free terminal amino groups in the solid-phase synthesis of peptides. Analytical Biochemistry. 34(2):595–598. doi:10.1016/0003-2697(70)90146-6

Information Classification: General

*Table SI 1. Analytical data for compounds **4-15**. Gradient applied: 30-70% B in A in 5'.*

COMPOUND	*SEQUENCE*	*RETENTION TIME [min]*	*m/z calc.* $[M_w+1H]^+$	*m/z exp.* $[M_w+1H]^+$
**4**	Ac-FFY-OH	2.70	518.6	518.31
**5**	Ac-FYY-OH	1.42	534.6	534.32
**6**	Ac-FWY-OH	2.55	557.6	557.36
**7**	TSC**1**-FFY-OH	3.40	681.2	681.49
**8***	TSC**1**-FYY-OH	3.80	697.2	697.8
**9**	TSC**1**-FWY-OH	3.53	720.2	720.34
**10**	TSC**2**-FFY-OH	3.62	785.3	785.60
**11**	TSC**2**-FYY-OH	2.95	801.3	801.51
**12**	TSC**2**-FWY-OH	3.52	824.3	824.60
**13**	TSC**3**-FFY-OH	3.40	695.3	695.47
**14**	TSC**3**-FYY-OH	2.68-93	711.3	711.46
**15**	TSC**3**-FWY-OH	3.45-68	734.3	734.47

*gradient applied: 20-70% of B in A in 5'.

Information Classification: General

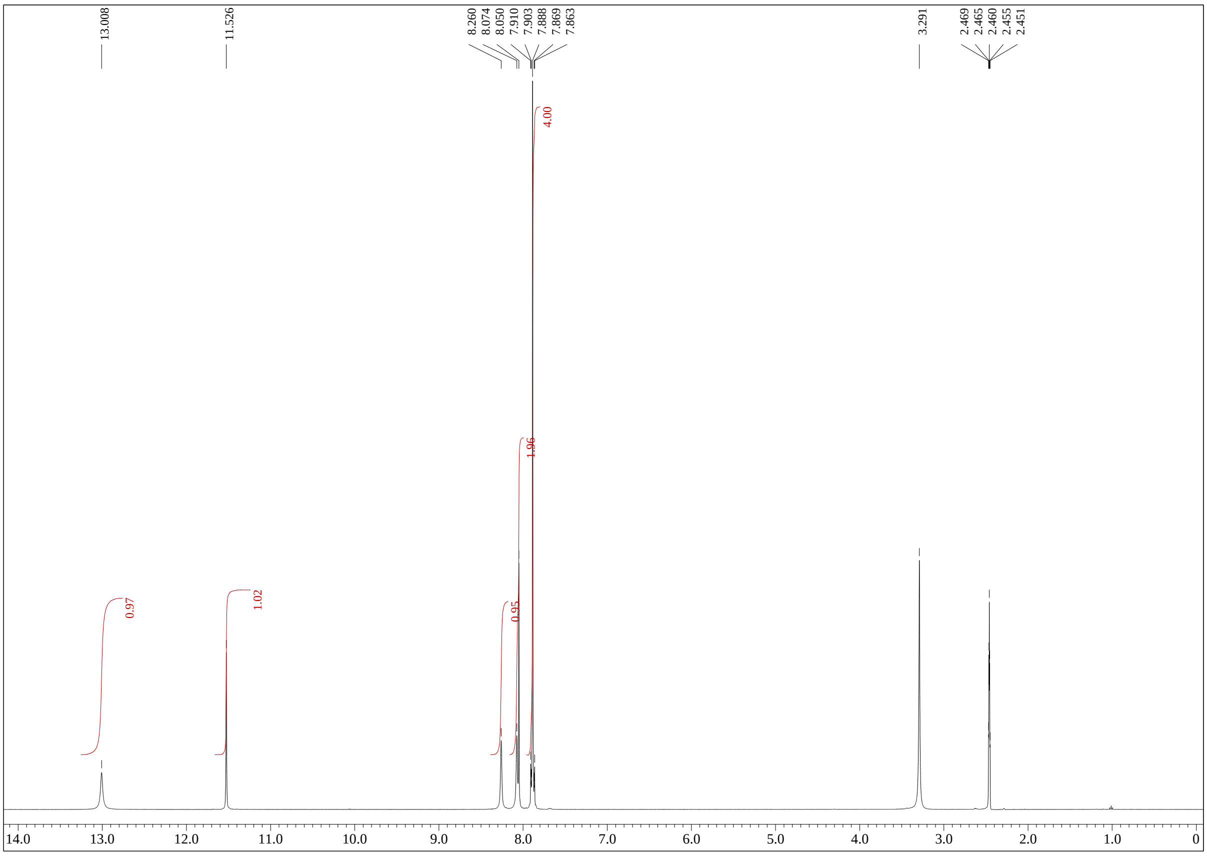

Figure SI 1. *^{1}H NMR spectrum of TSC* ***1****.*

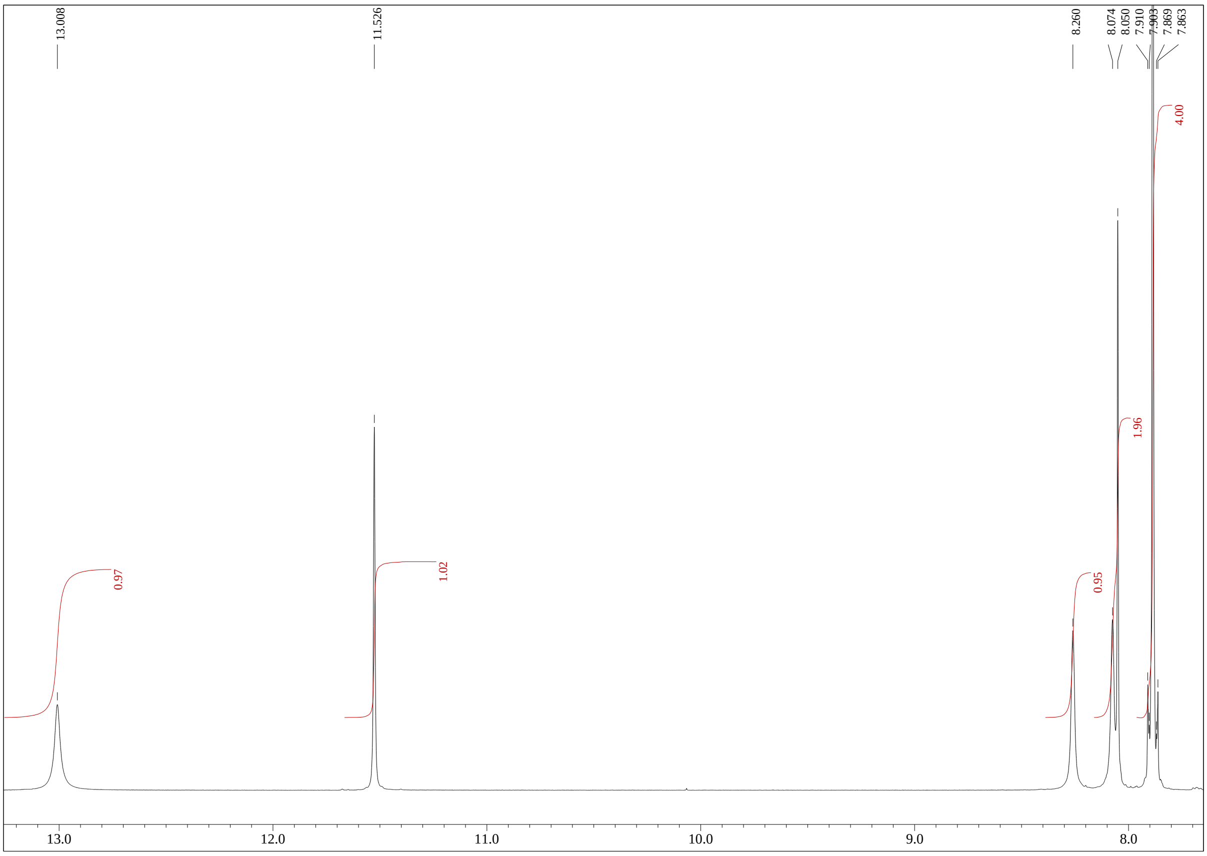

Figure SI 2. *Expanded ^{1}H NMR spectrum of TSC* ***1****.*

Information Classification: General

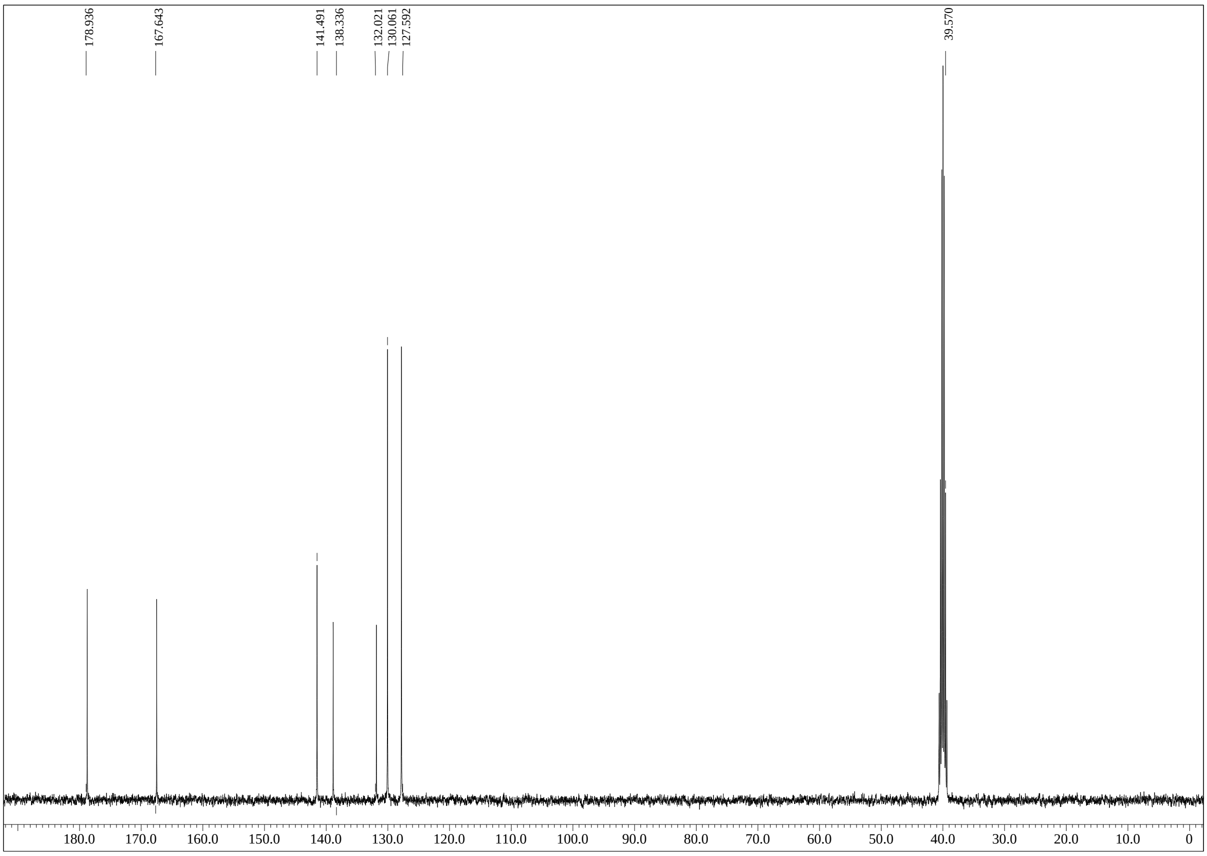

Figure SI 3. *$^{13}C\{^1H\}$ NMR spectrum of TSC **1**.*

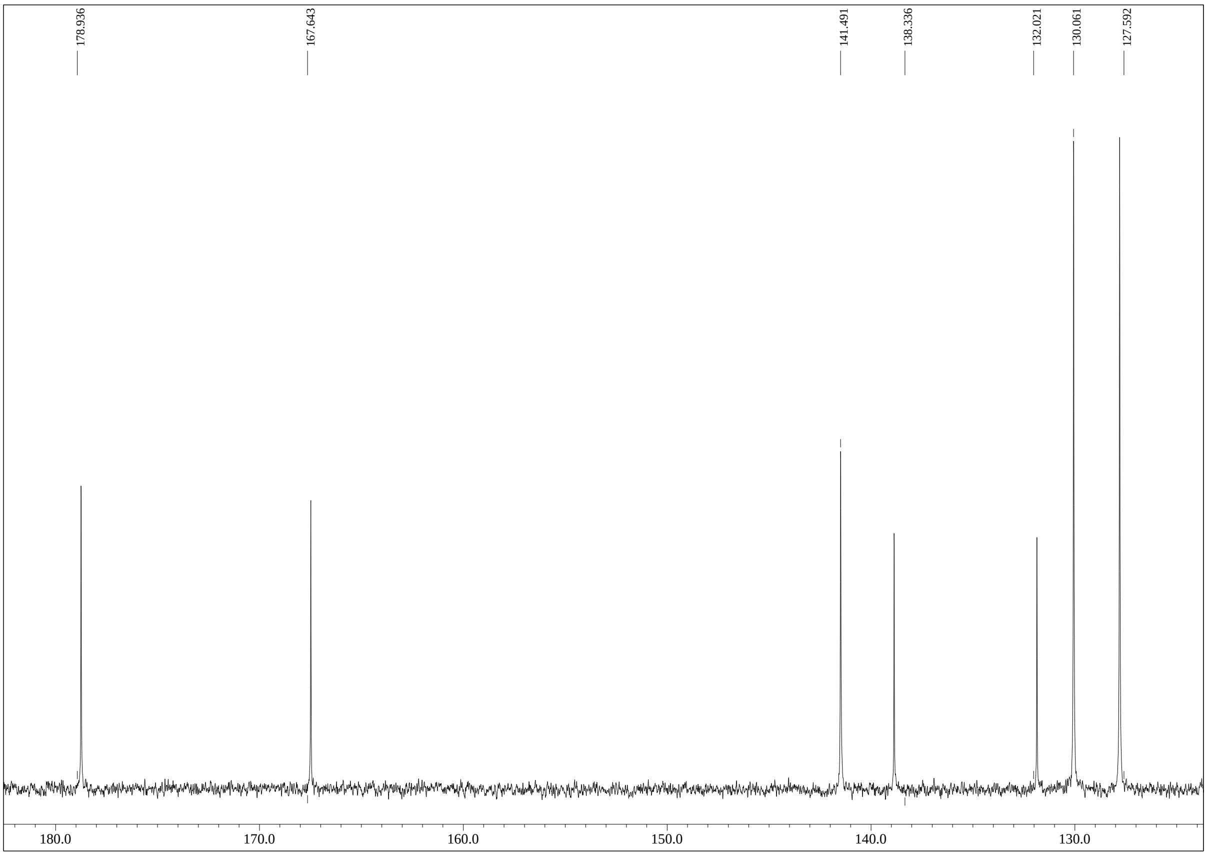

Figure SI 4. *Expanded $^{13}C\{^1H\}$ NMR spectrum of TSC **1**.*

Information Classification: General

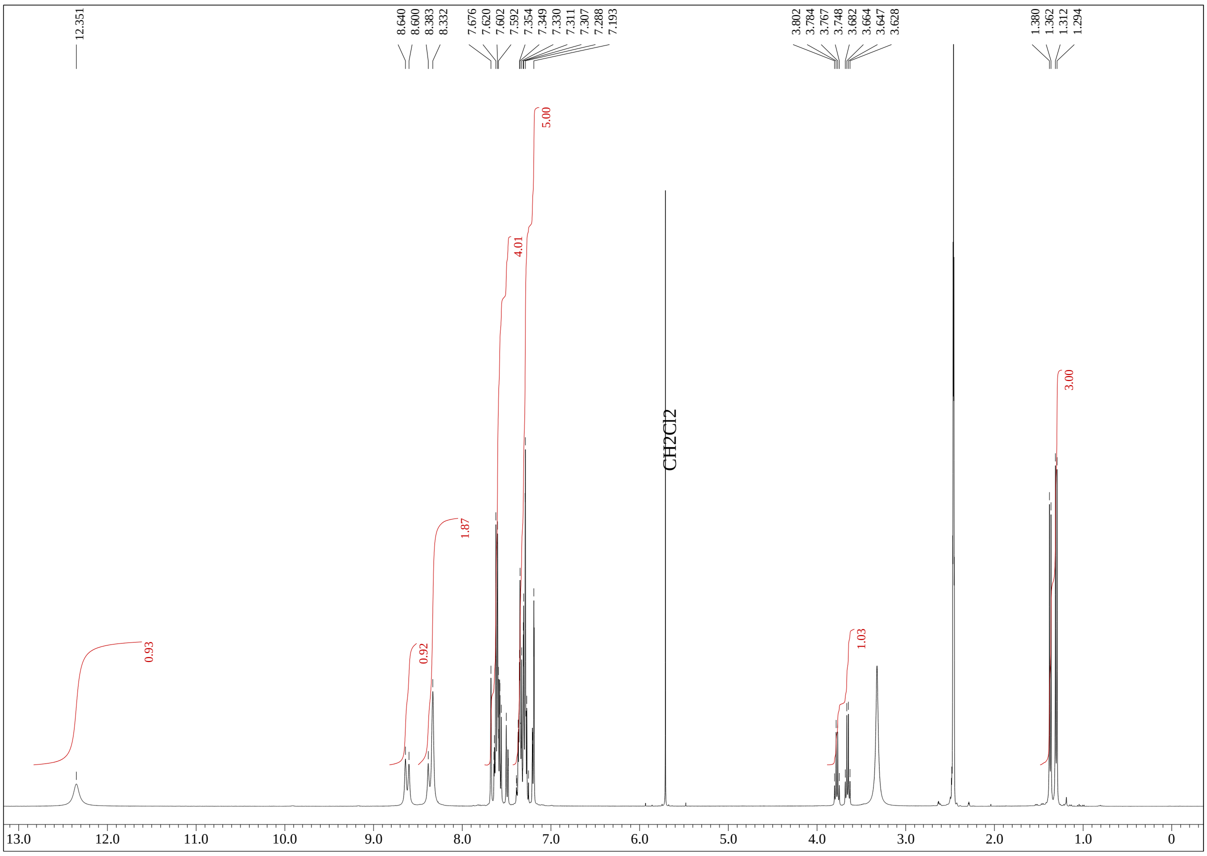

Figure SI 5. *^{1}H NMR spectrum of TSC **2**.*

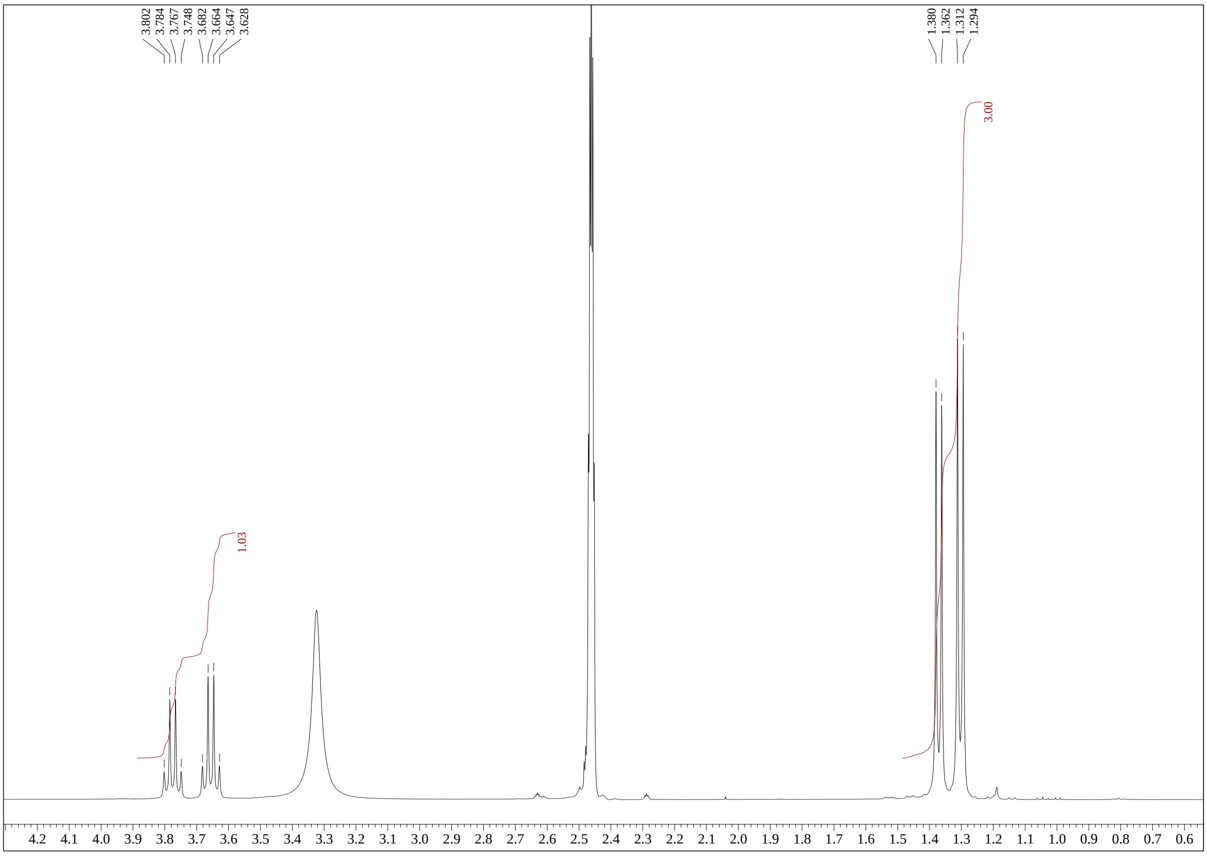

Figure SI 6. *Expanded ^{1}H NMR spectrum of TSC **2**.*

Information Classification: General

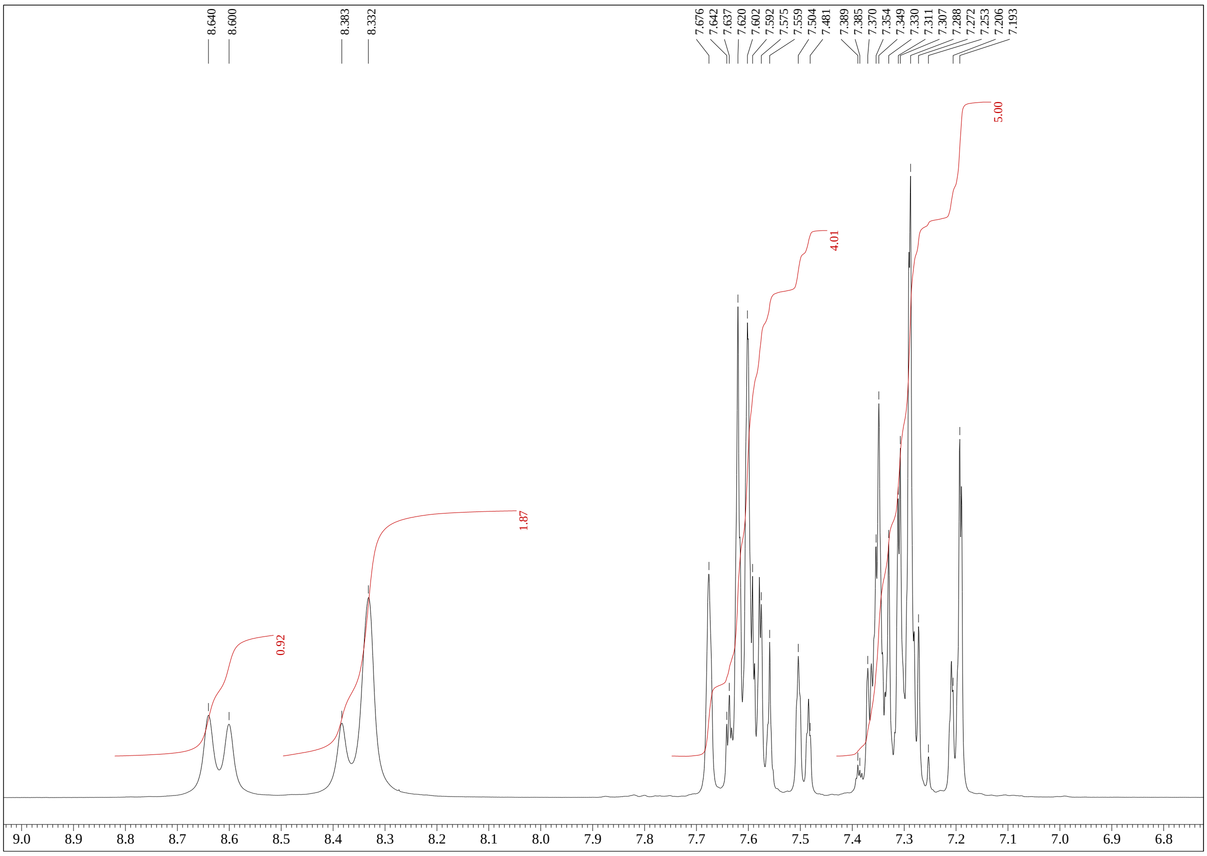

Figure SI 7. *Expanded* ^{1}H *NMR spectrum of TSC* ***2****.*

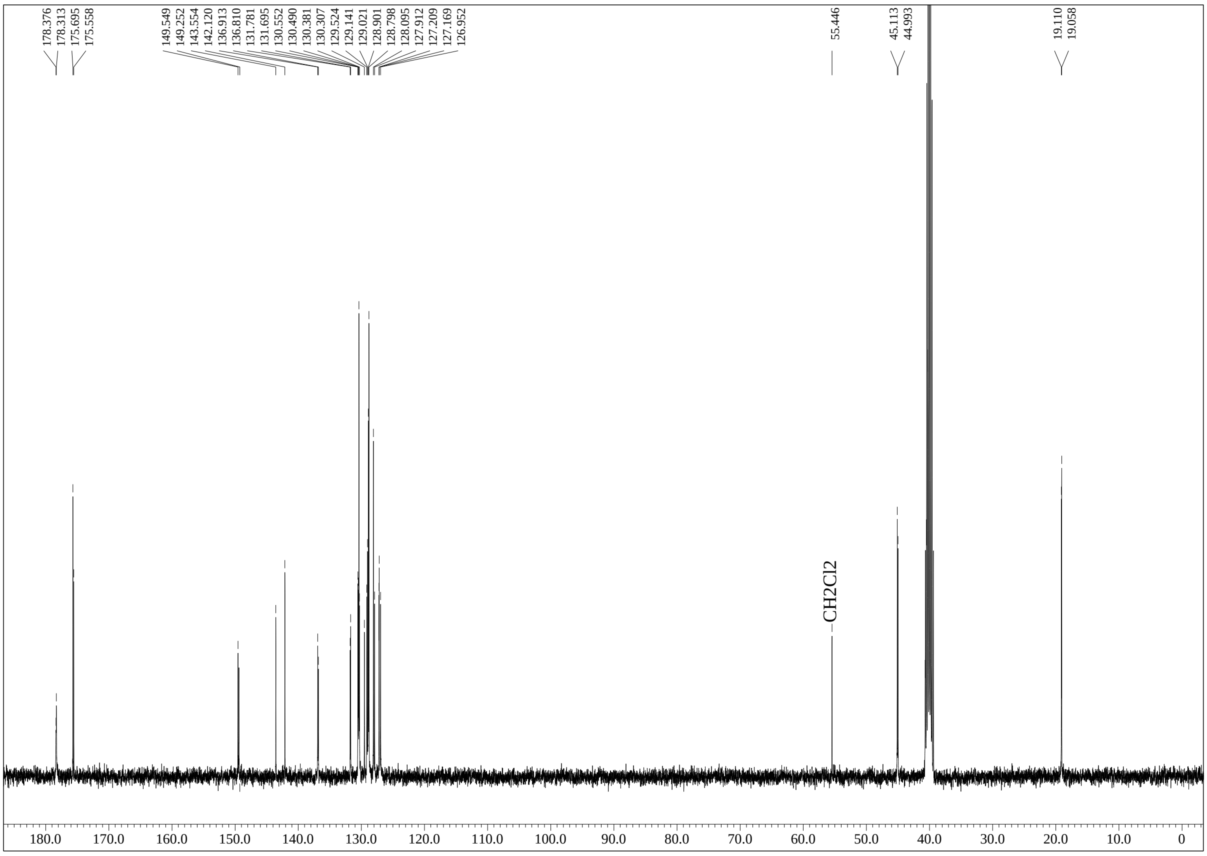

Figure SI 8. $^{13}C\{^{1}H\}$ *NMR spectrum of TSC* ***2****.*

Information Classification: General

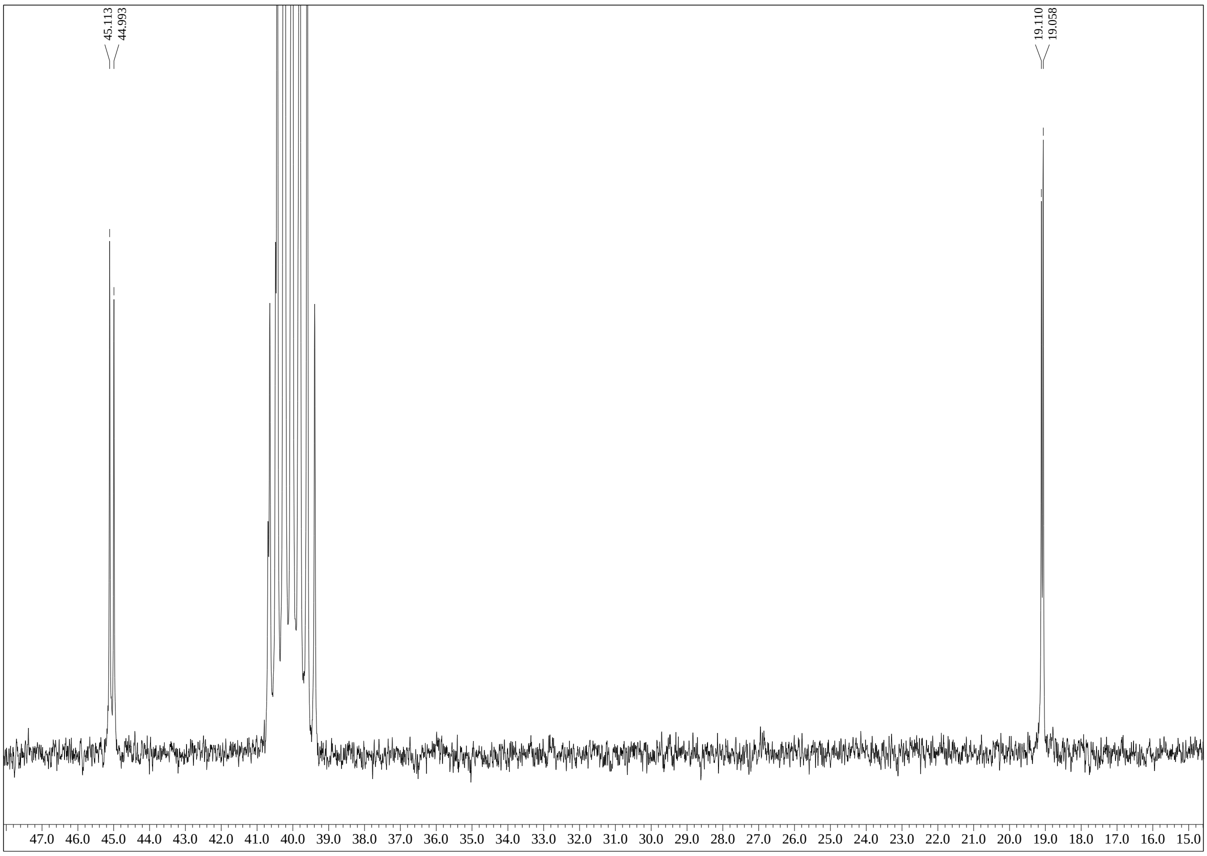

Figure SI 9. *Expanded* $^{13}C\{^{1}H\}$ *NMR spectrum of TSC* ***2****.*

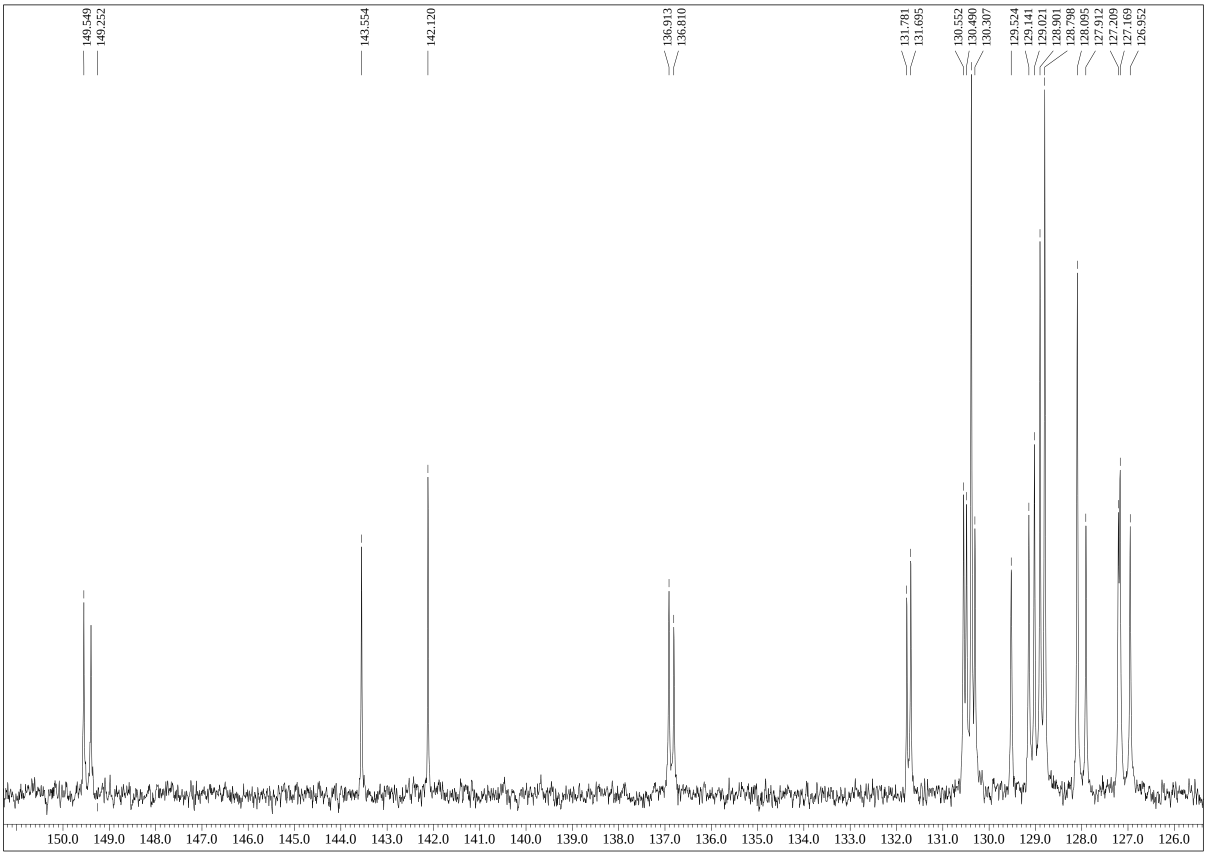

Figure SI 10. *Expanded* $^{13}C\{^{1}H\}$ *NMR spectrum of TSC* ***2****.*

Information Classification: General

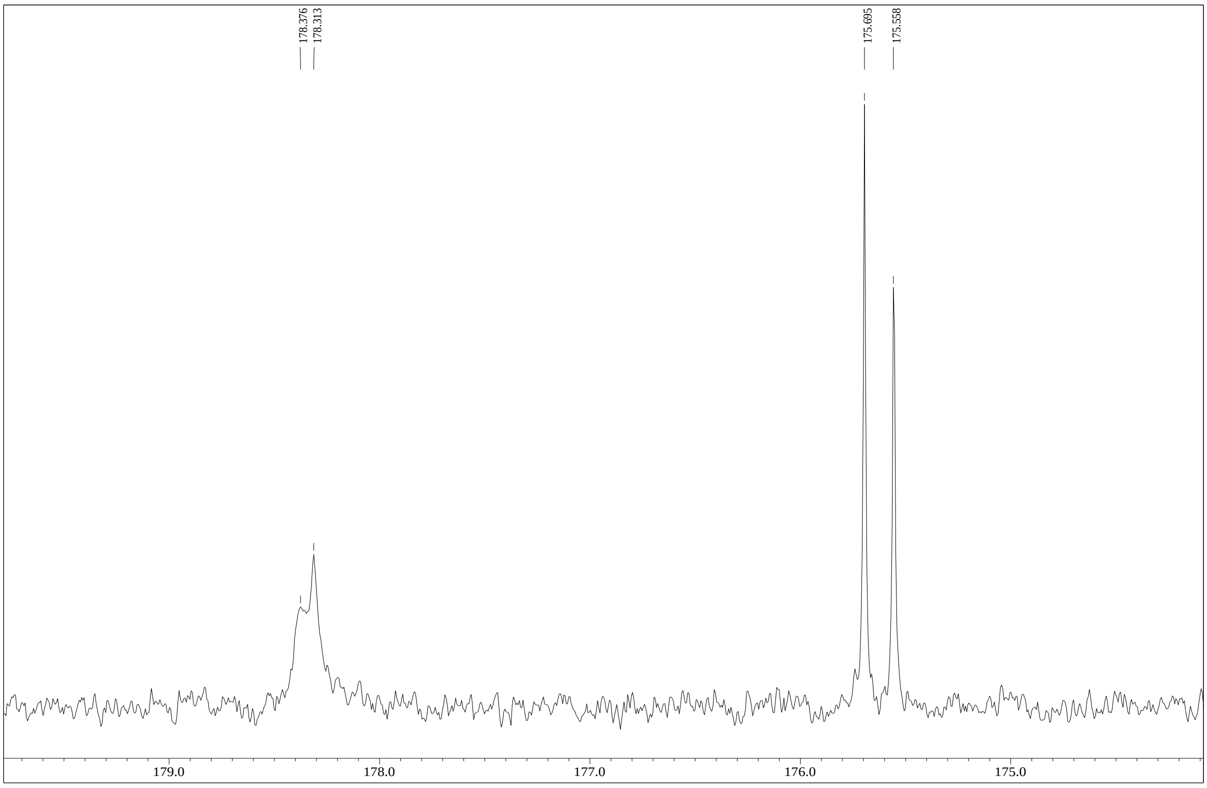

Figure SI 11. *Expanded $^{13}C\{^1H\}$ NMR spectrum of TSC **2**.*

Information Classification: General

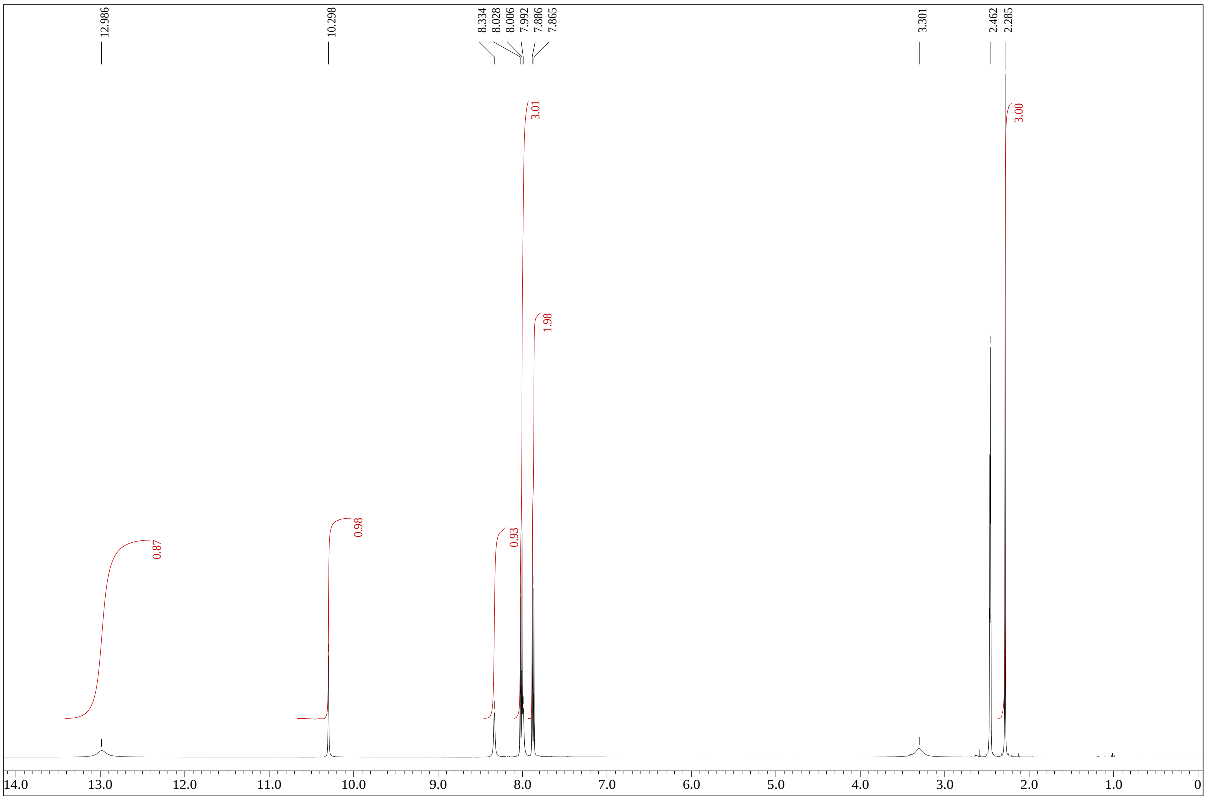

Figure SI 12. *^{1}H NMR spectrum of TSC* ***3***.

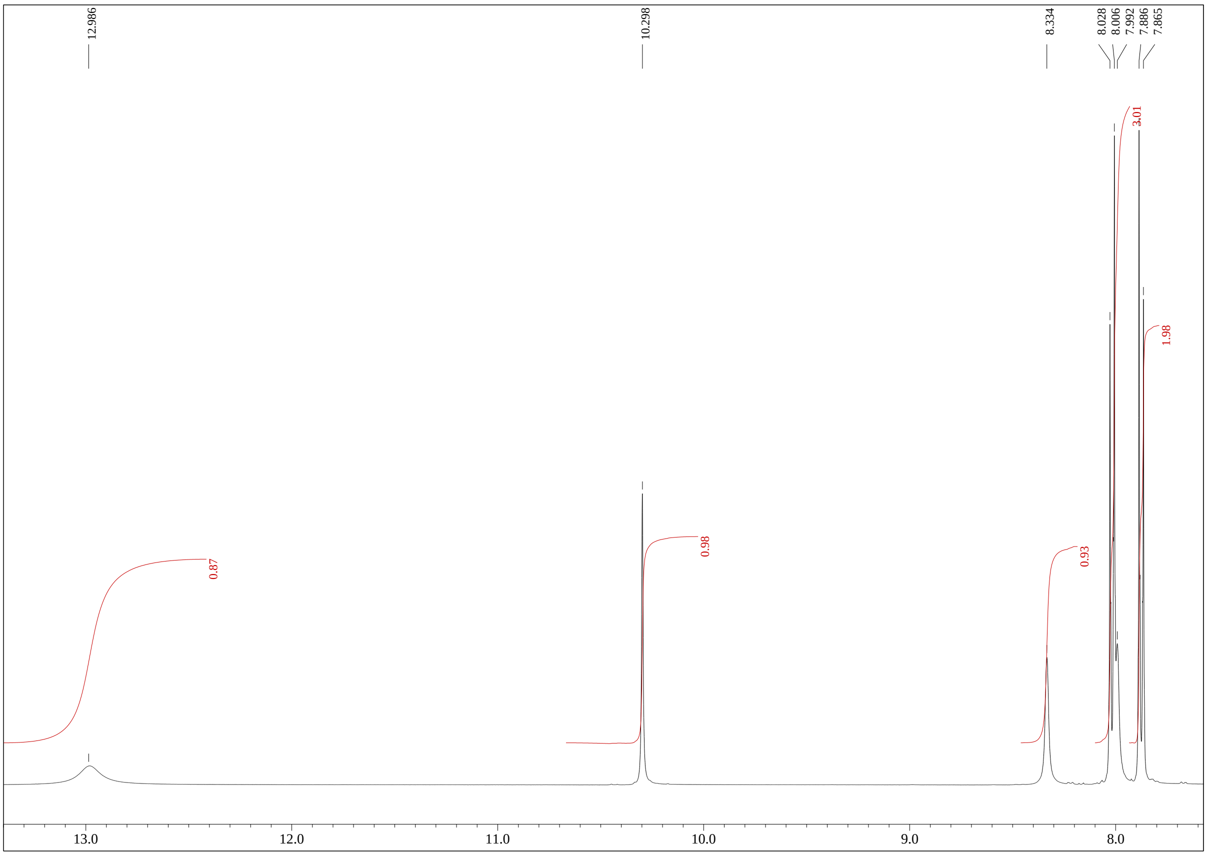

Figure SI 13. *Expanded ^{1}H NMR spectrum of TSC* ***3***.

Information Classification: General

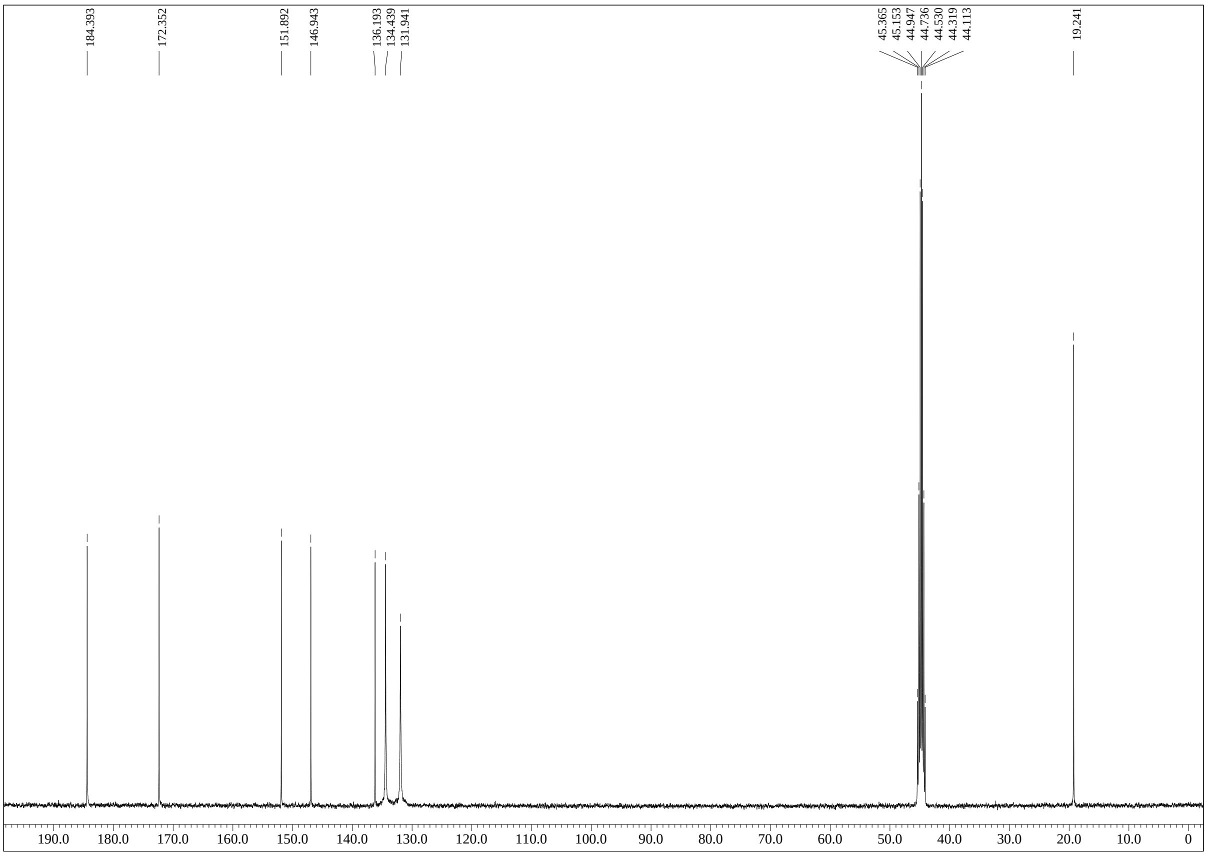

Figure SI 14. *$^{13}C\{^{1}H\}$ NMR spectrum of TSC **3**.*

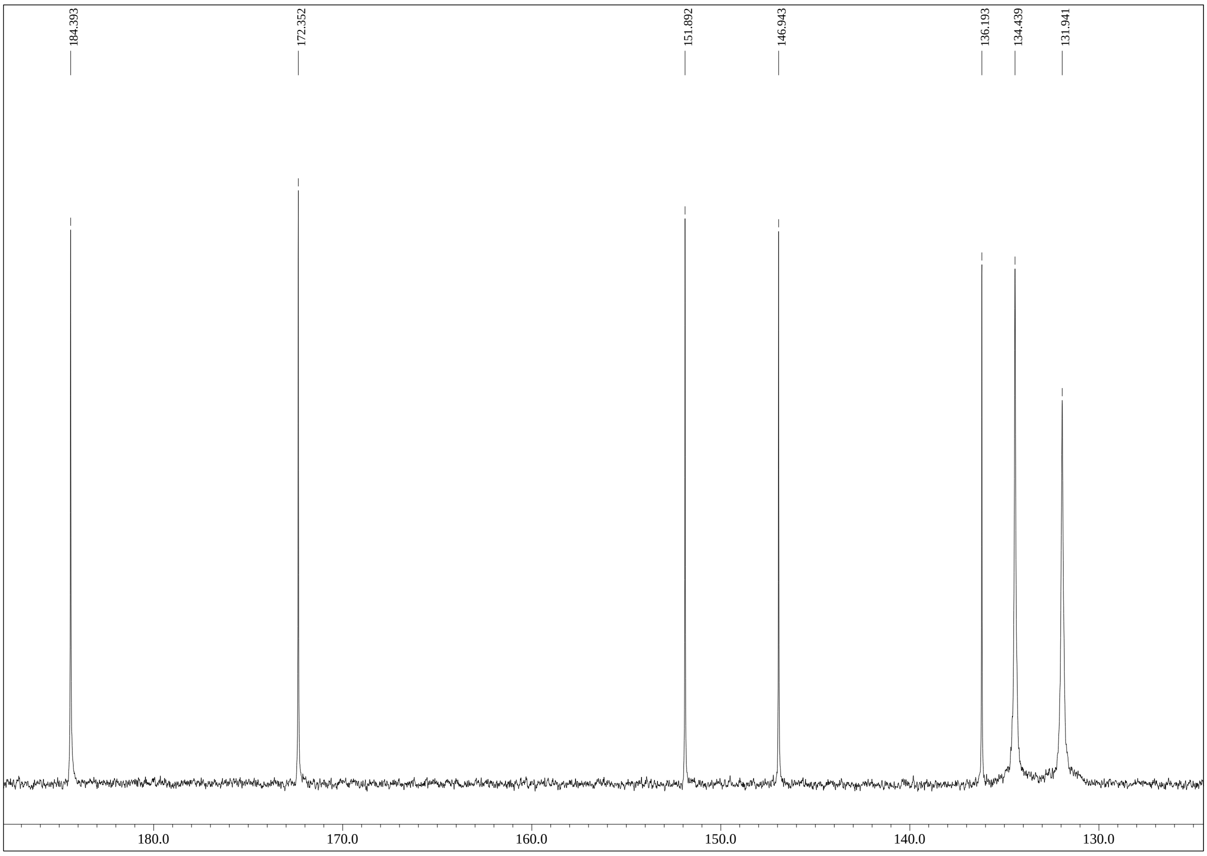

Figure SI 15. *Expanded $^{13}C\{^{1}H\}$ NMR spectrum of TSC **3**.*

Information Classification: General

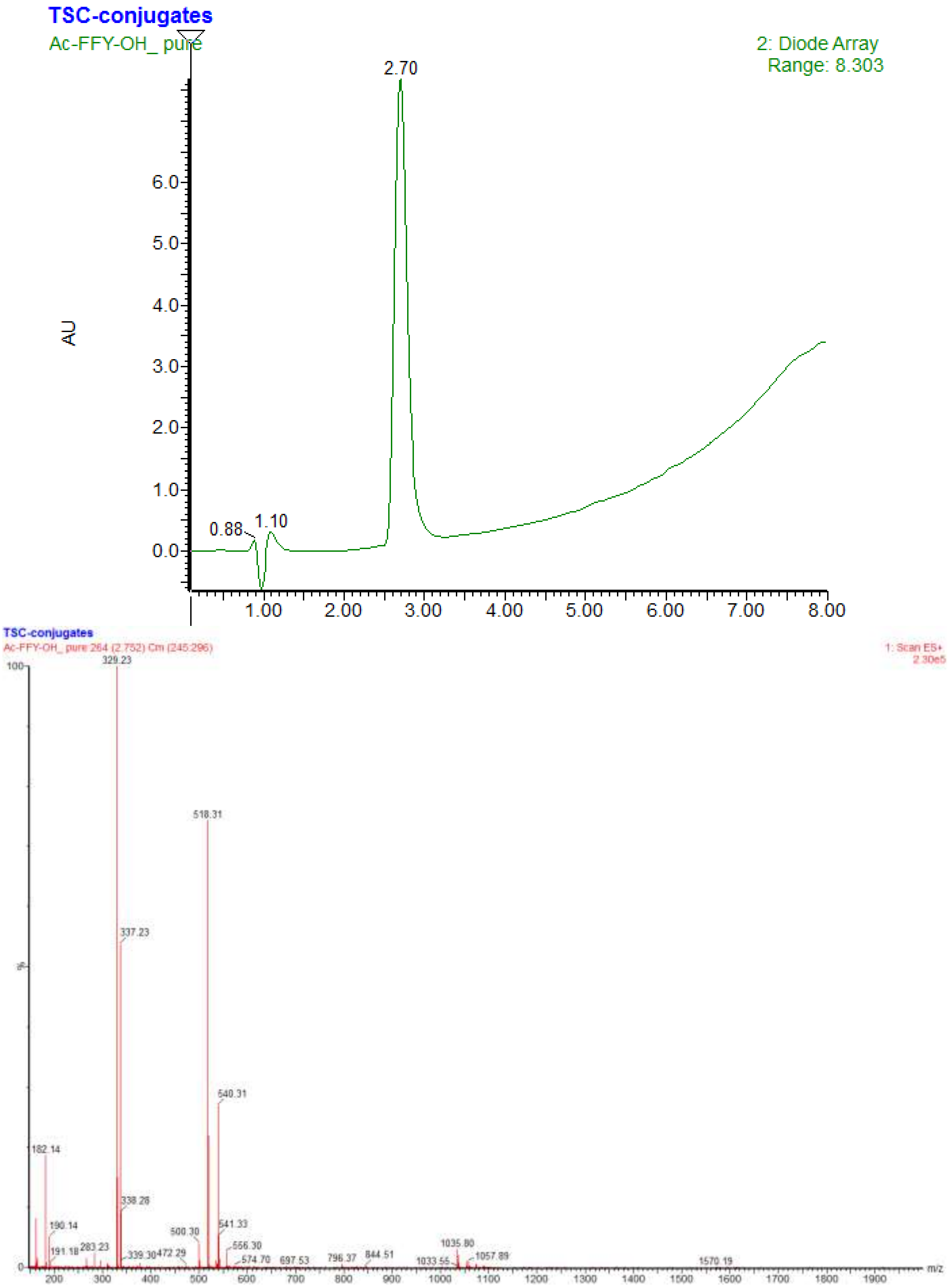

Figure SI 16. *Chromatogram of* ***4*** *(Ac-FFY-OH) and MS spectra of the peak 2.7 min.*

Information Classification: General

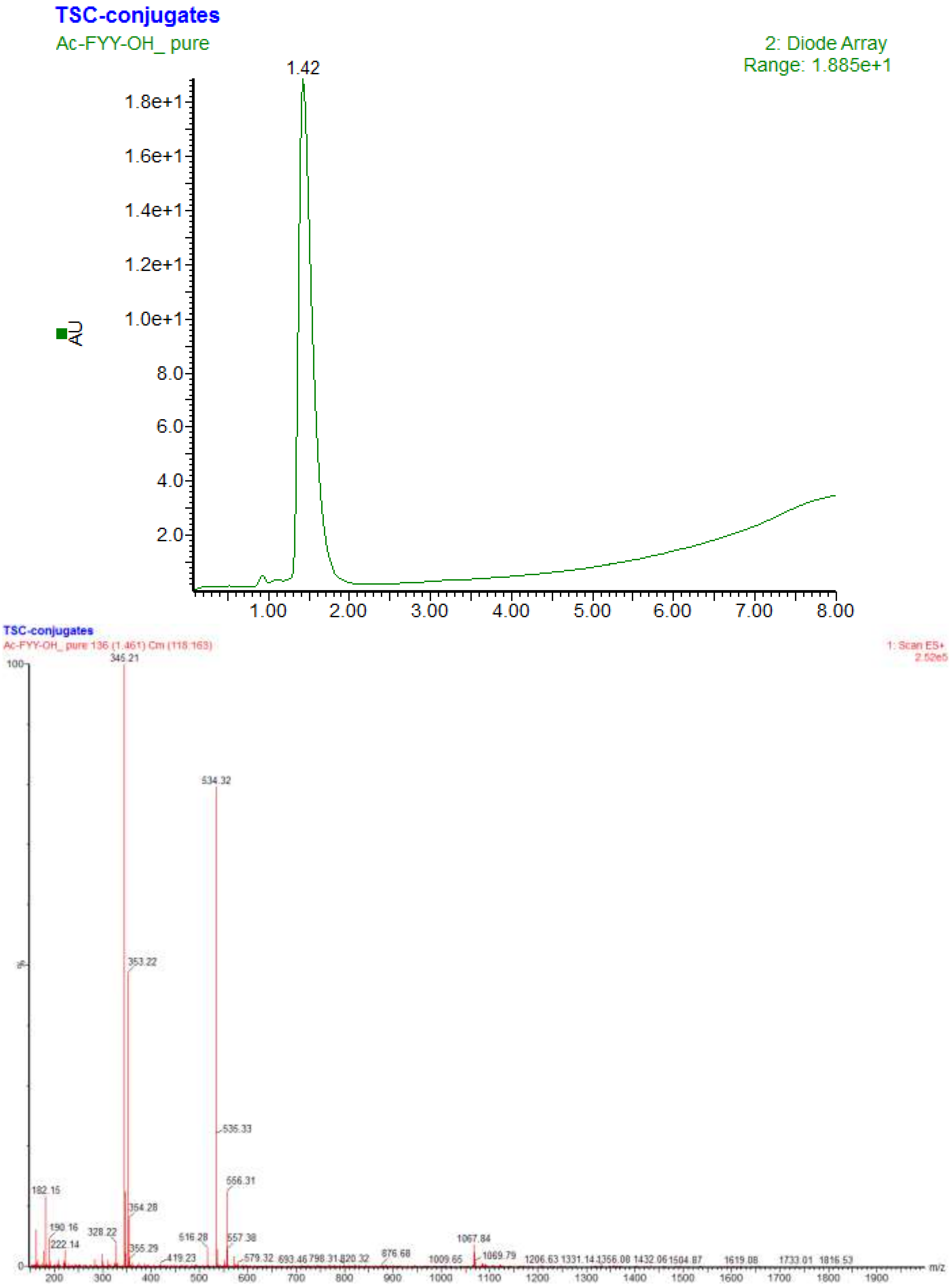

Figure SI 17. *Chromatogram of* ***5*** *(Ac-FYY-OH) and MS spectra of the peak 1.4 min.*

Information Classification: General

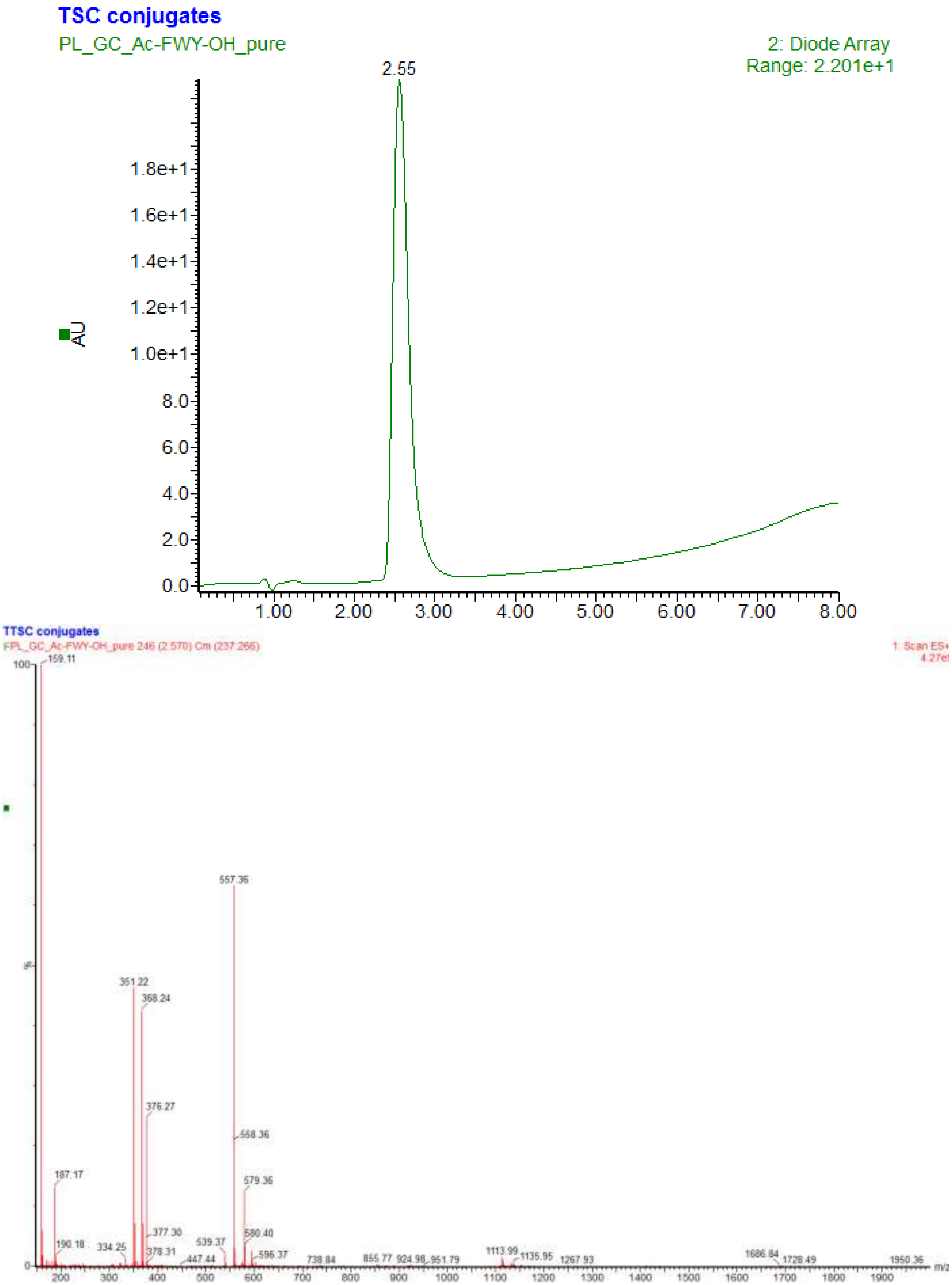

Figure SI 18. *Chromatogram of* ***6*** *(Ac-FWY-OH) and MS spectra of the peak 2.5 min.*

Information Classification: General

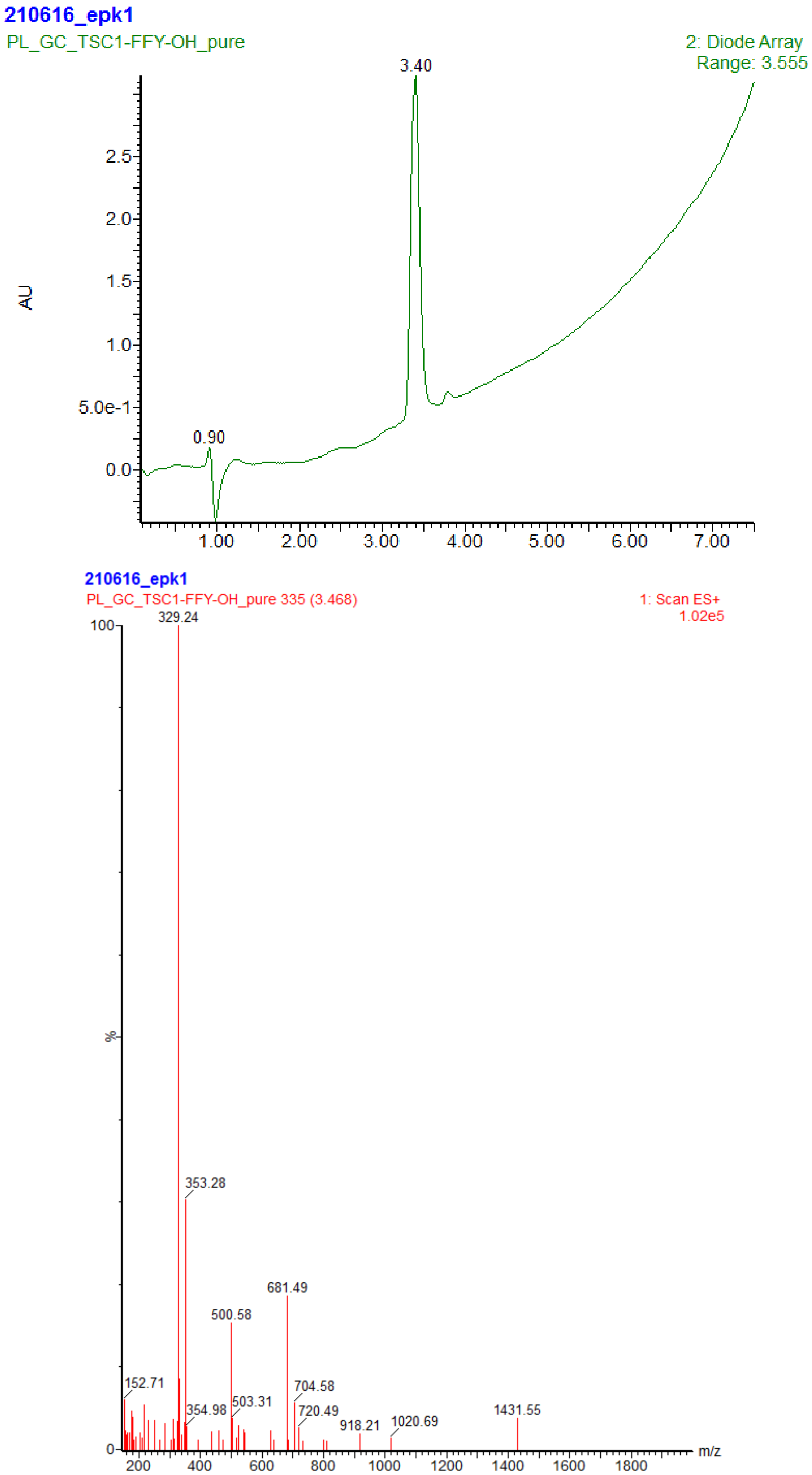

Figure SI 19. *Chromatogram of **7** and MS spectra of the peak 3.4 min.*

Information Classification: General

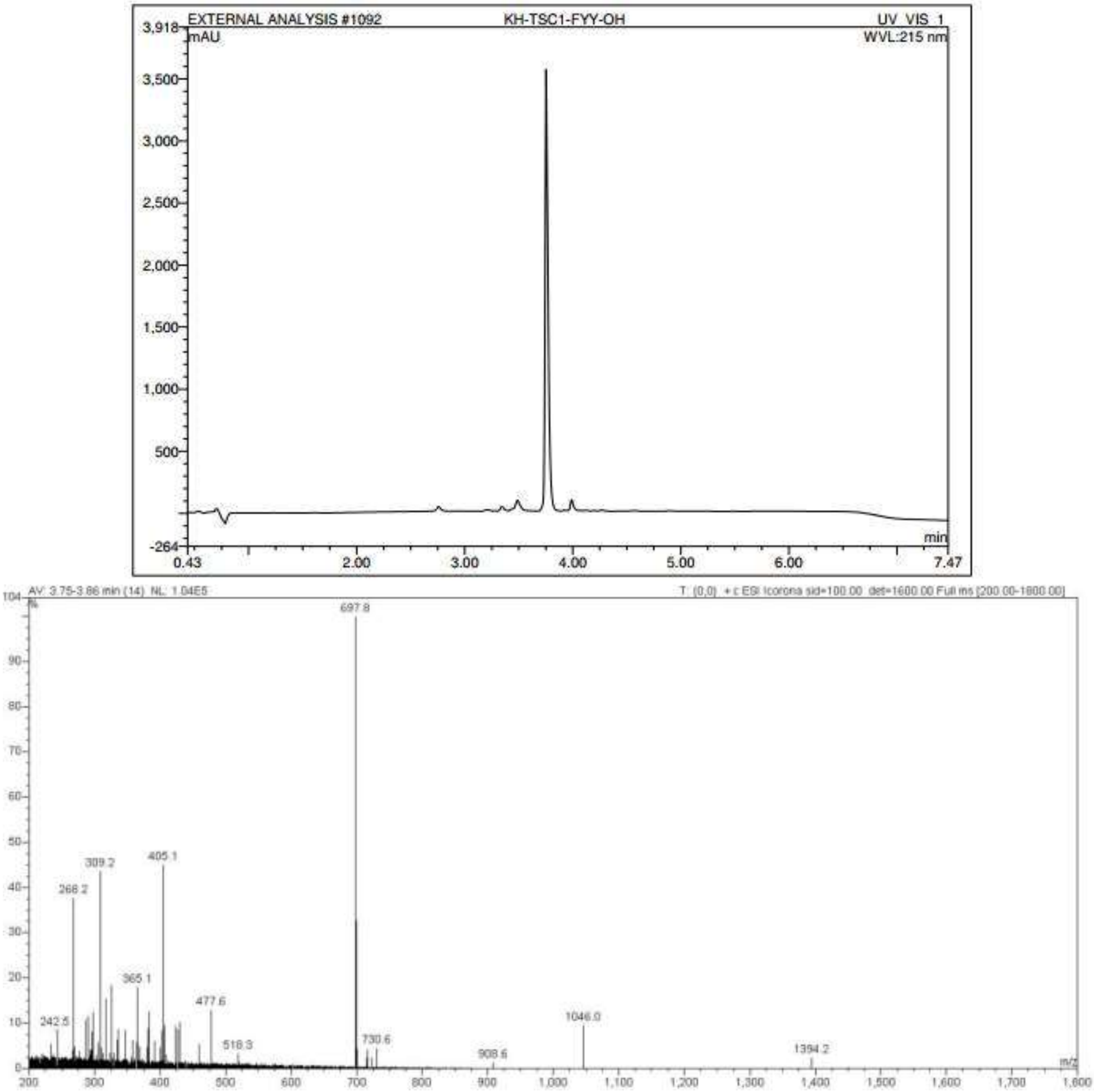

__Figure SI 20__. Chromatogram of __8__ and MS spectra of the peak 3.8 min.

Information Classification: General

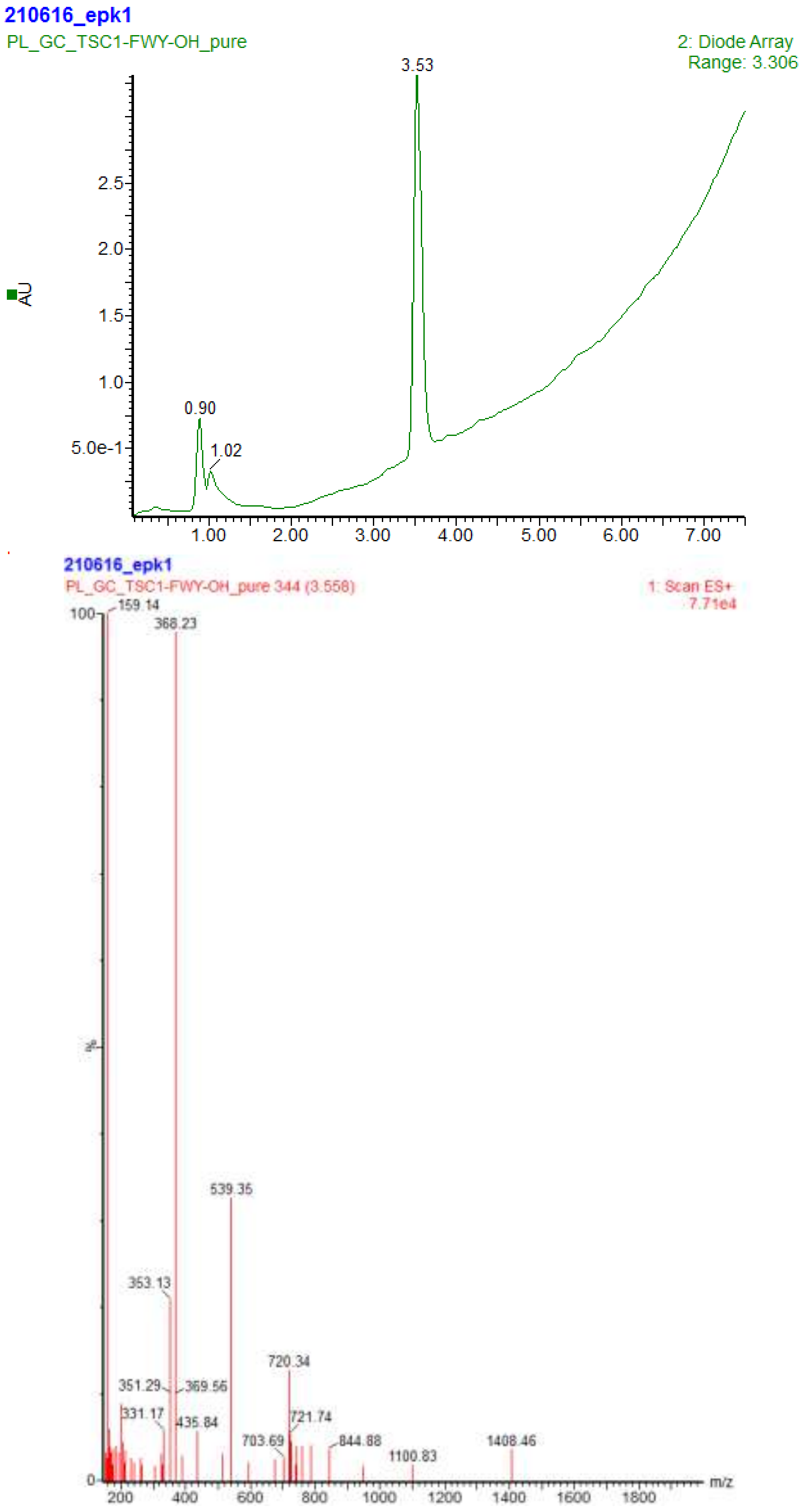

Figure SI 21. *Chromatogram of **9** and MS spectra of the peak 3.5 min.*

Information Classification: General

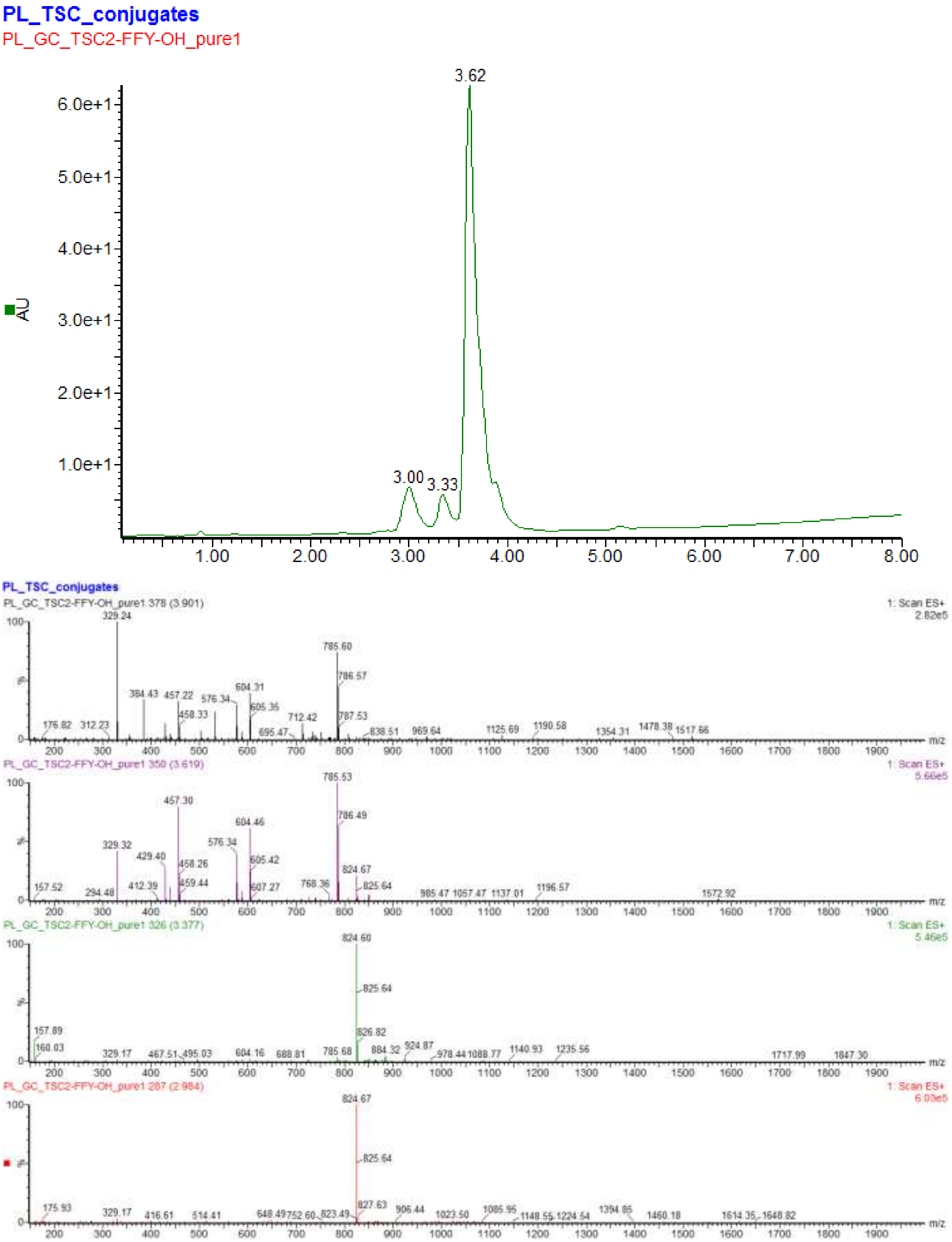

Figure SI 22. *Chromatogram of **10** and MS spectra of the peaks found.*

Information Classification: General

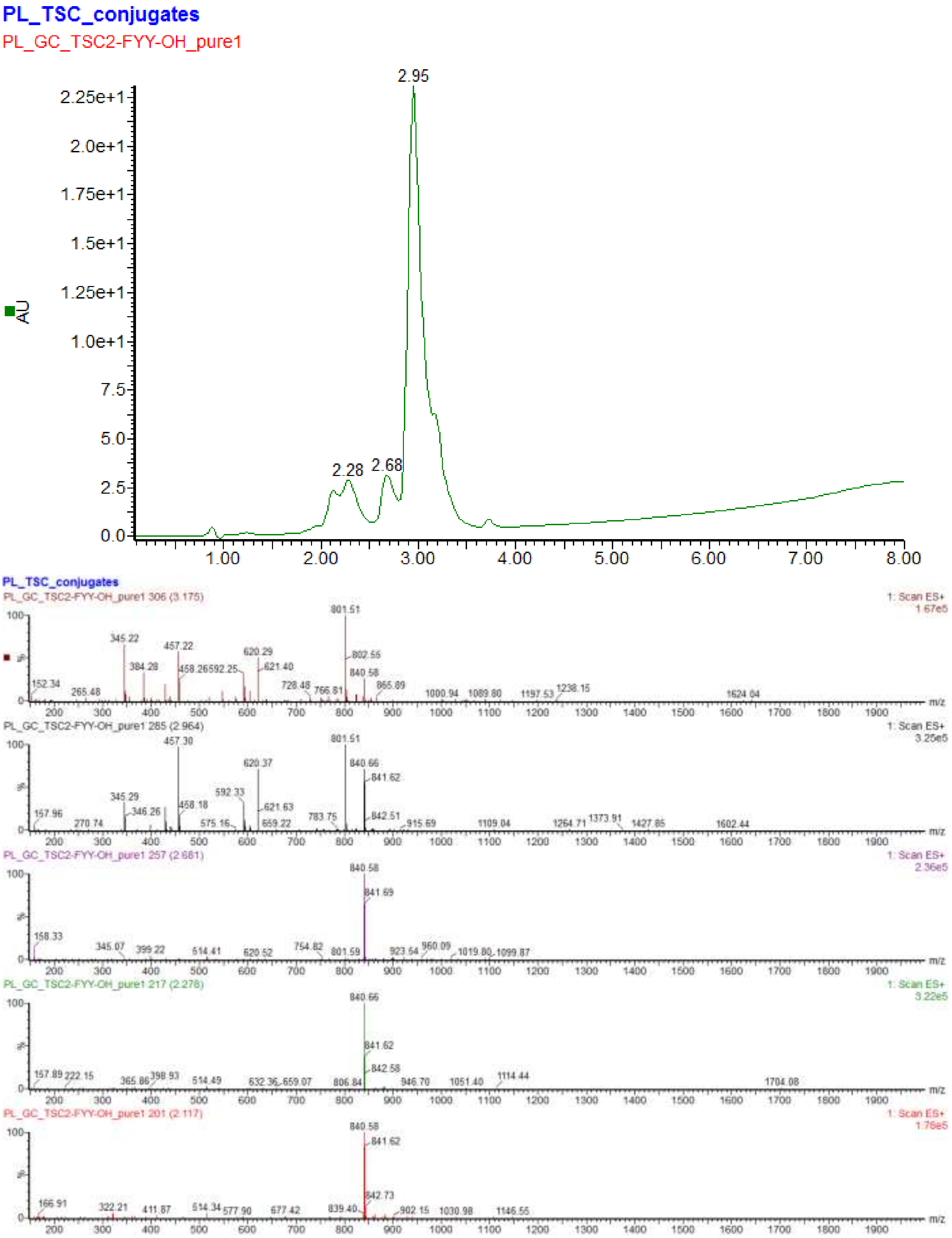

Figure SI 23. *Chromatogram of **11** and MS spectra of the peaks found.*

Information Classification: General

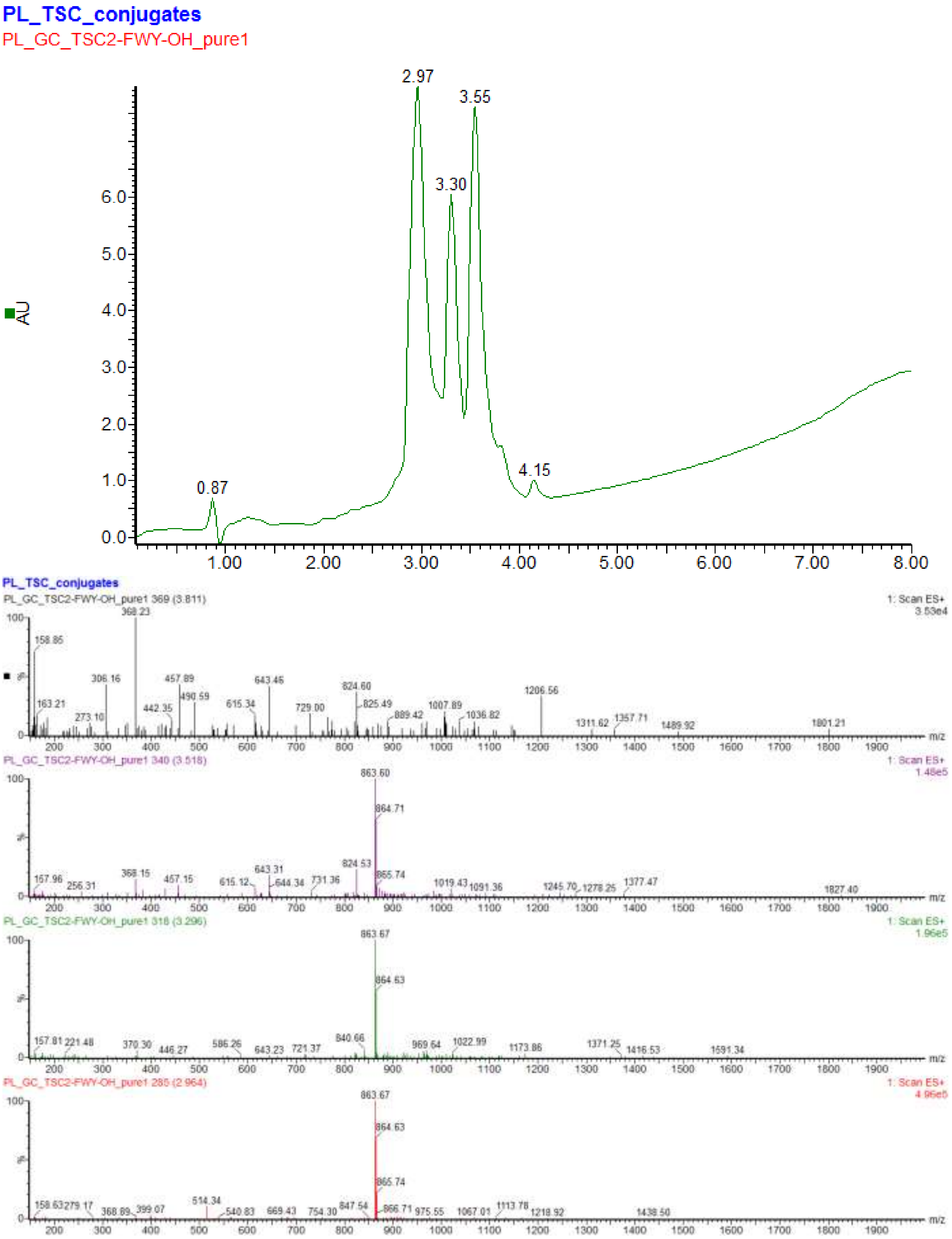

Figure SI 24. *Chromatogram of **12** and MS spectra of the peaks found.*

Information Classification: General

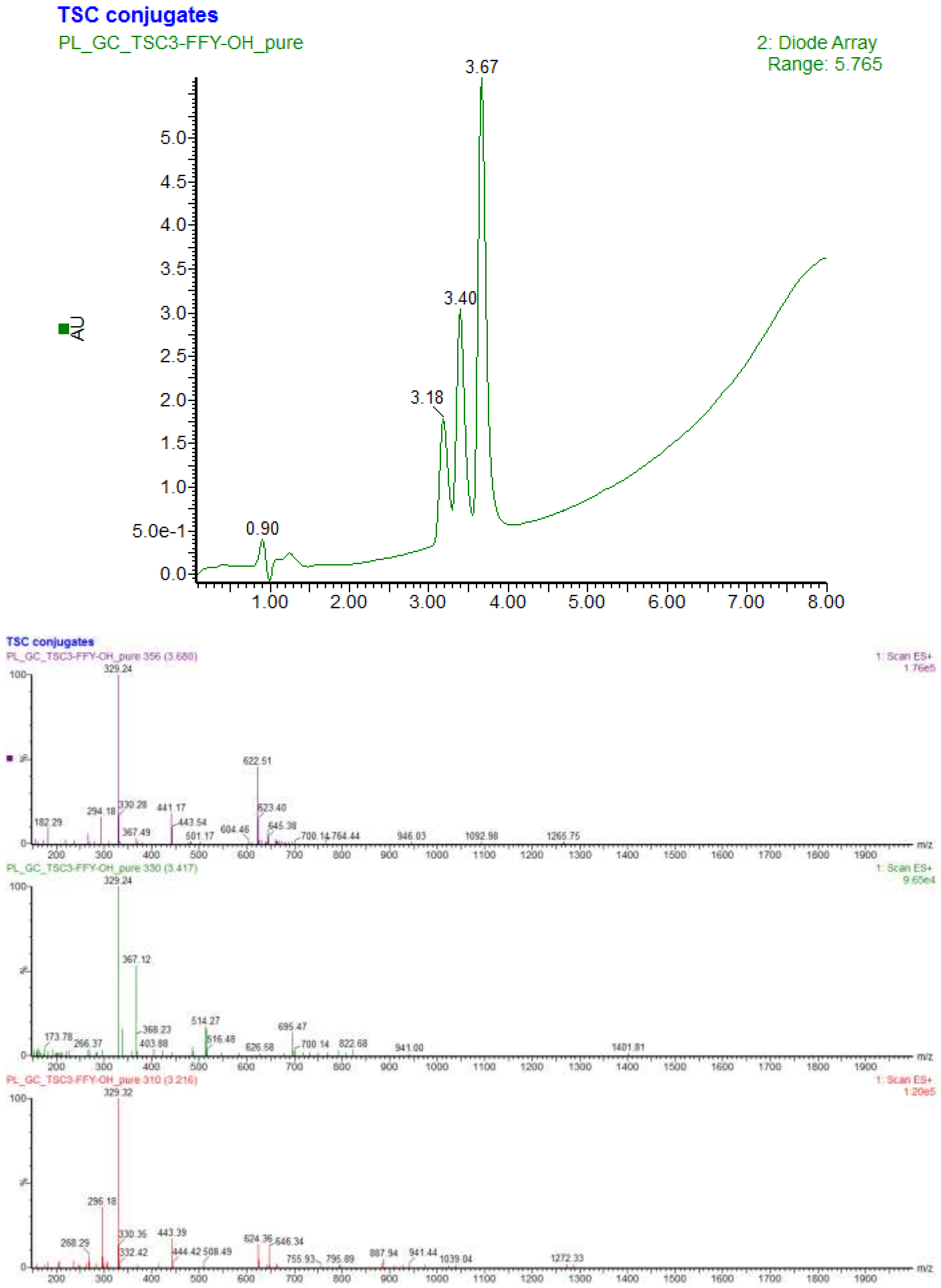

Figure SI 25. *Chromatogram of **13** and MS spectra of the peaks found.*

Information Classification: General

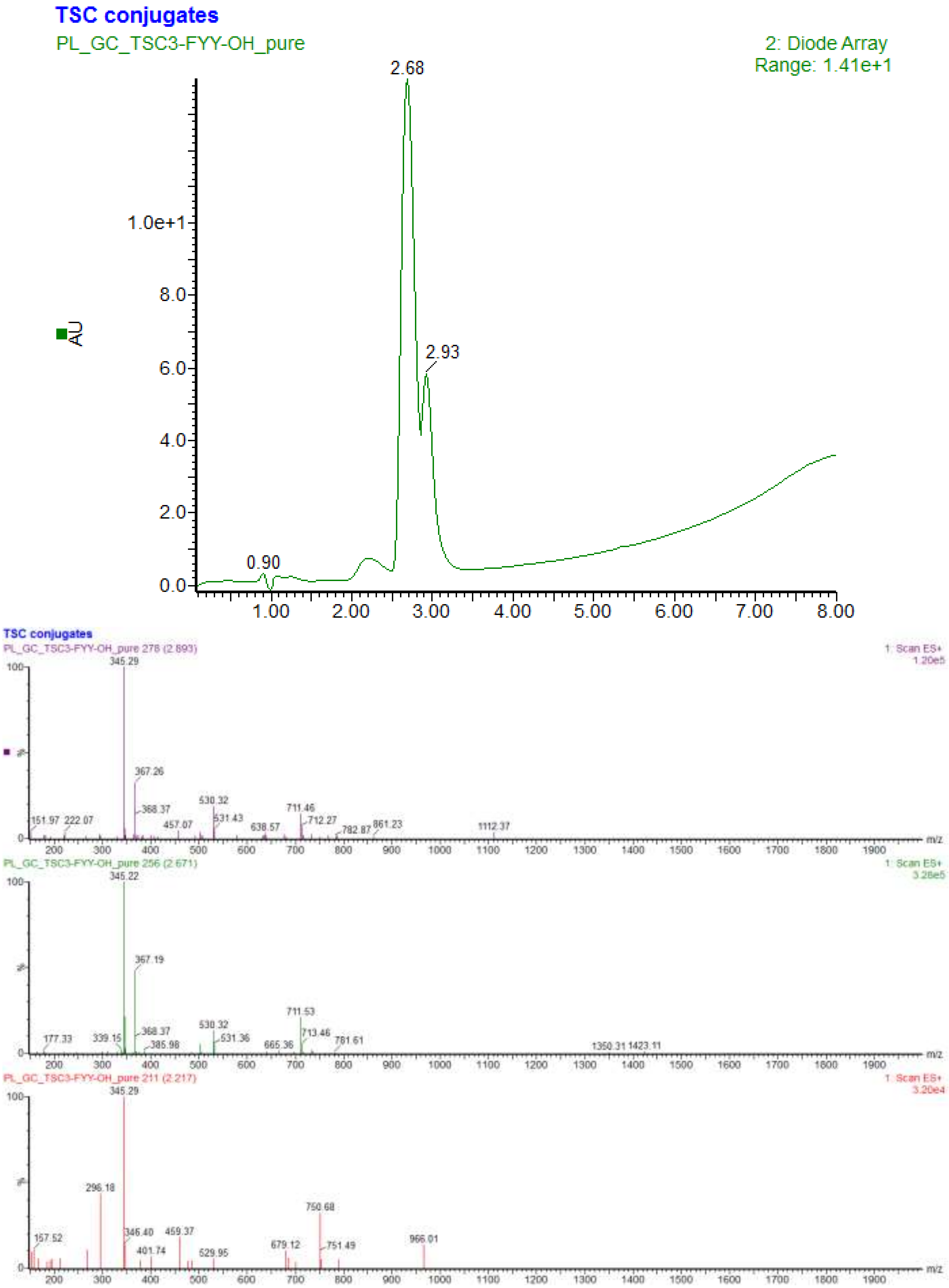

Figure SI 26. *Chromatogram of* ***14*** *and MS spectra of the peaks found.*

Information Classification: General

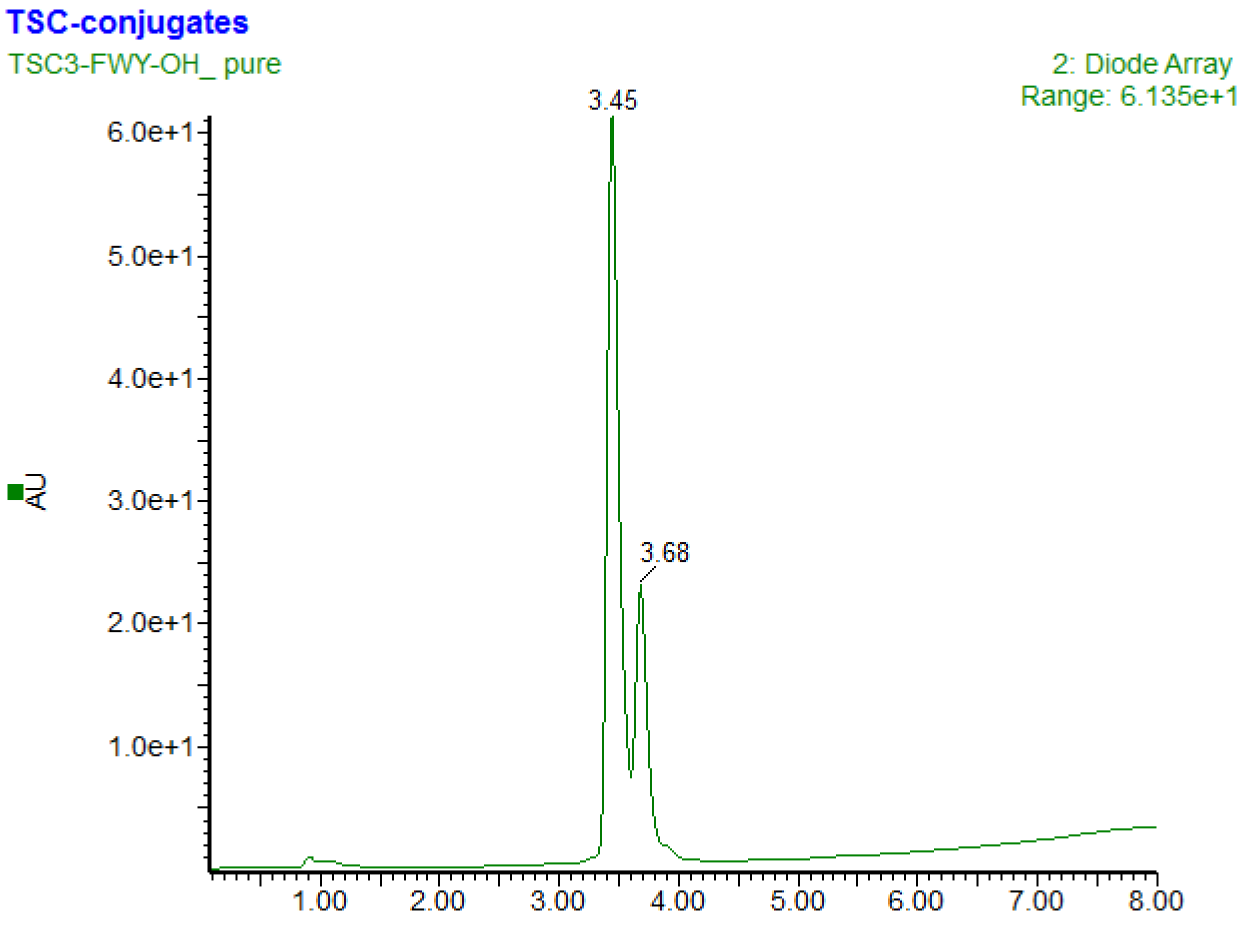

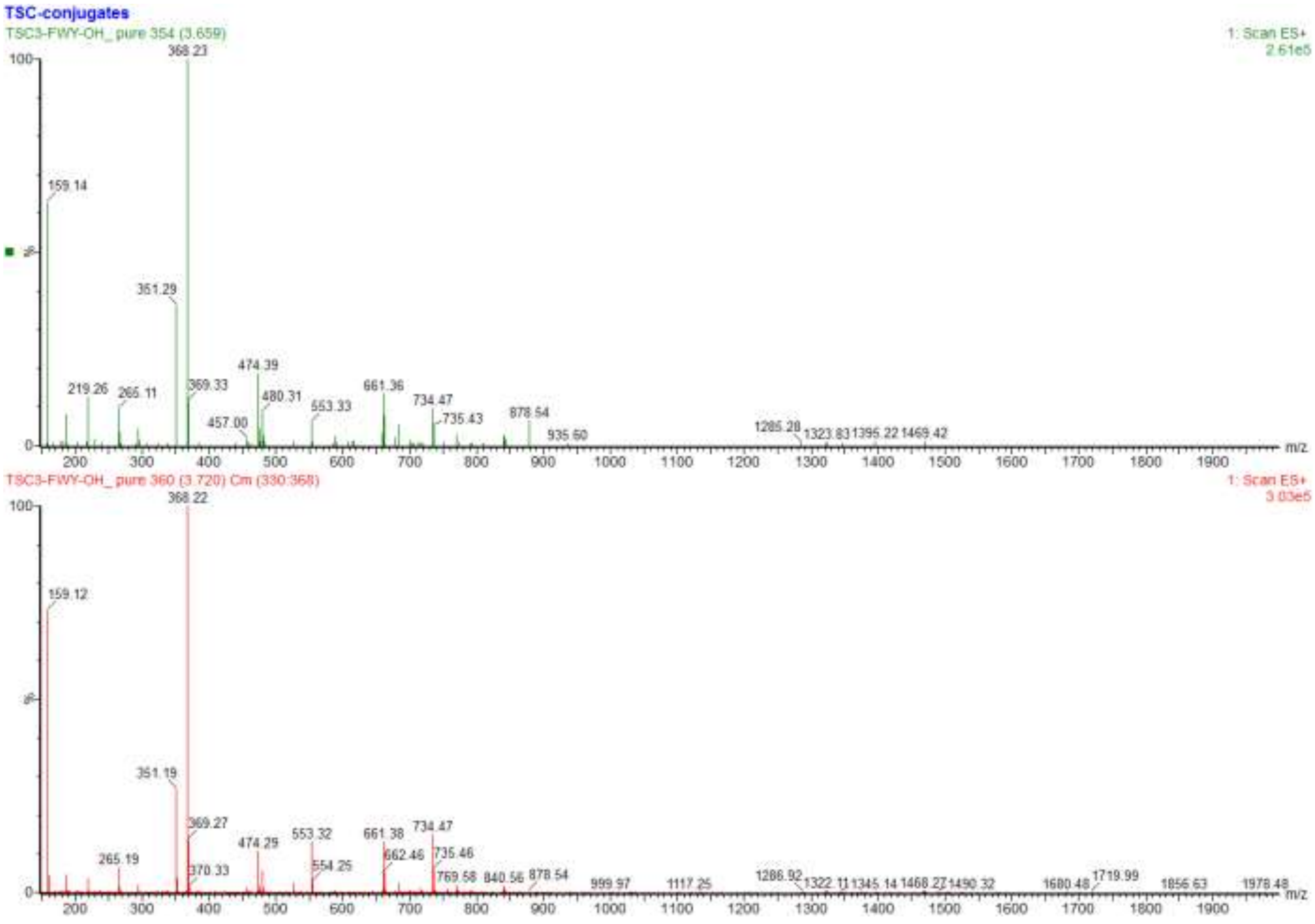

Information Classification: General

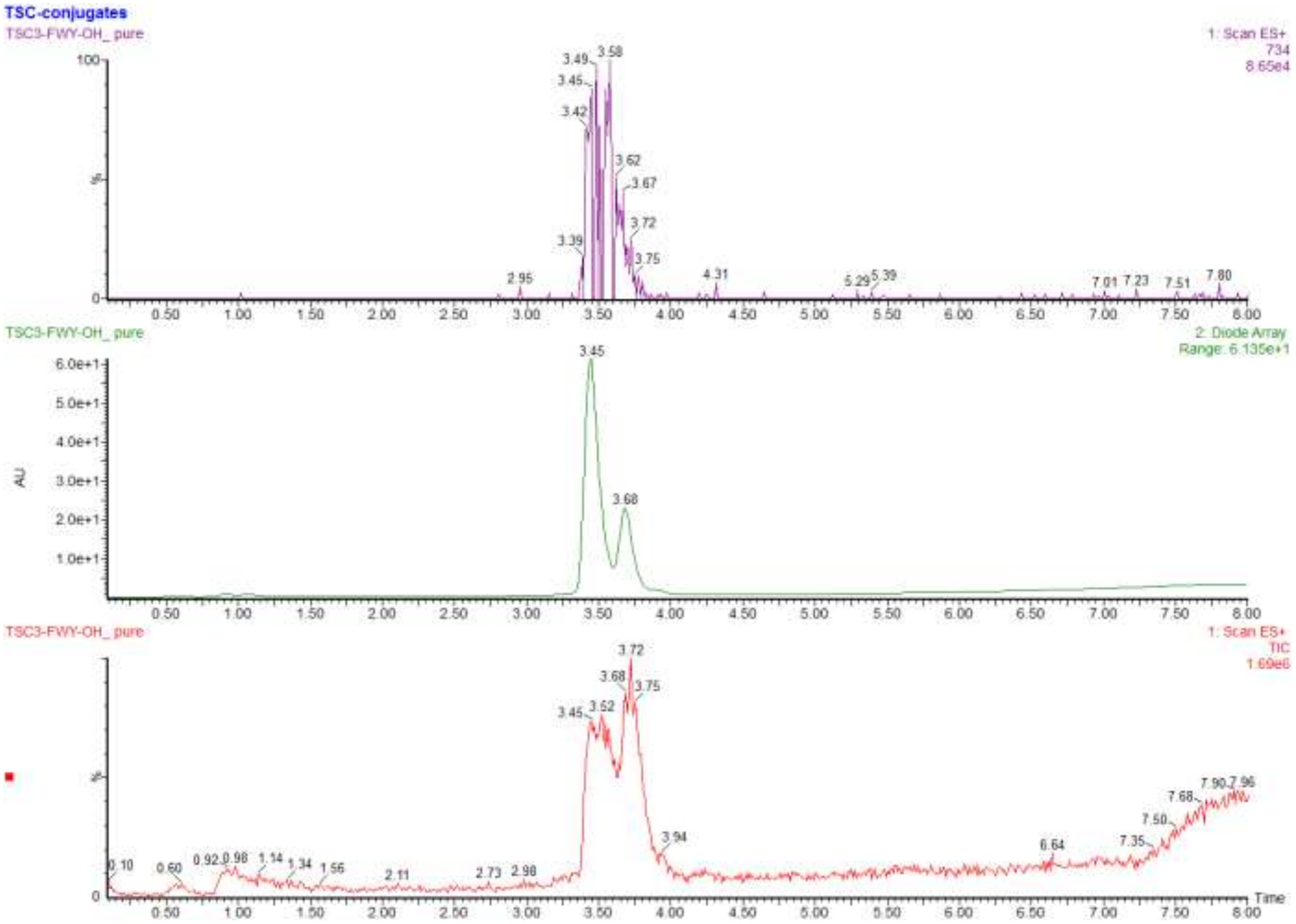

Figure SI 27. *Chromatogram of **15** and MS spectra of the peaks found.*

Information Classification: General

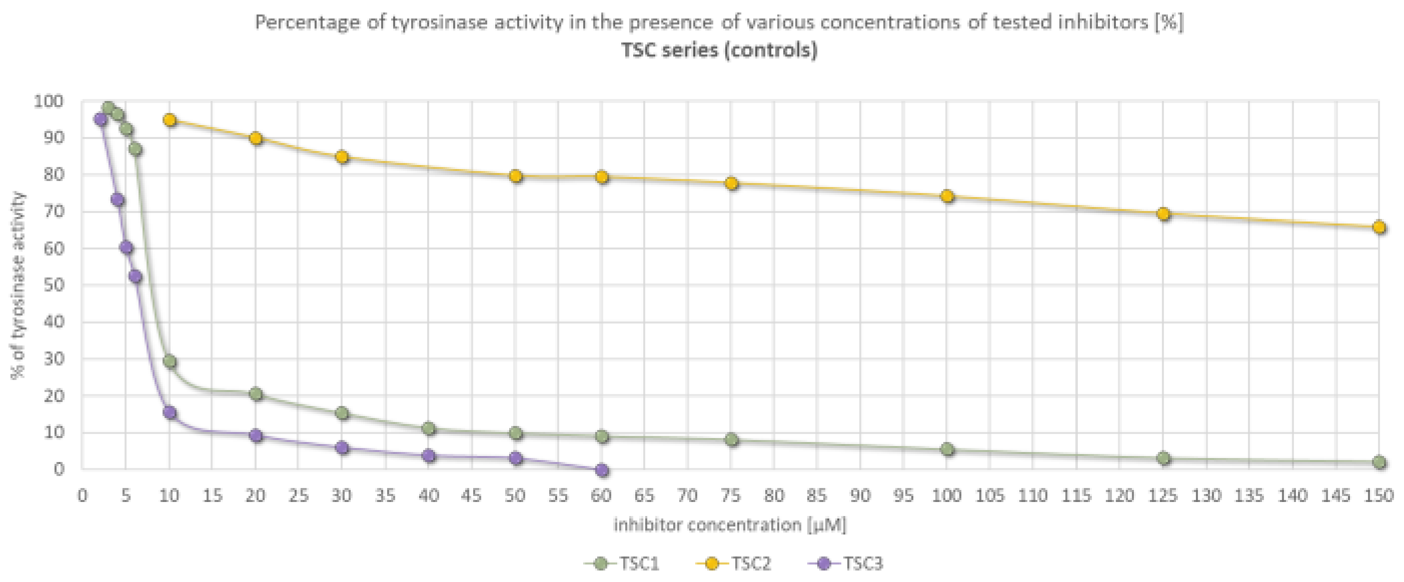

Figure SI 28. *Percentage of tyrosinase activity in the presence of compounds* ***1-3****.*

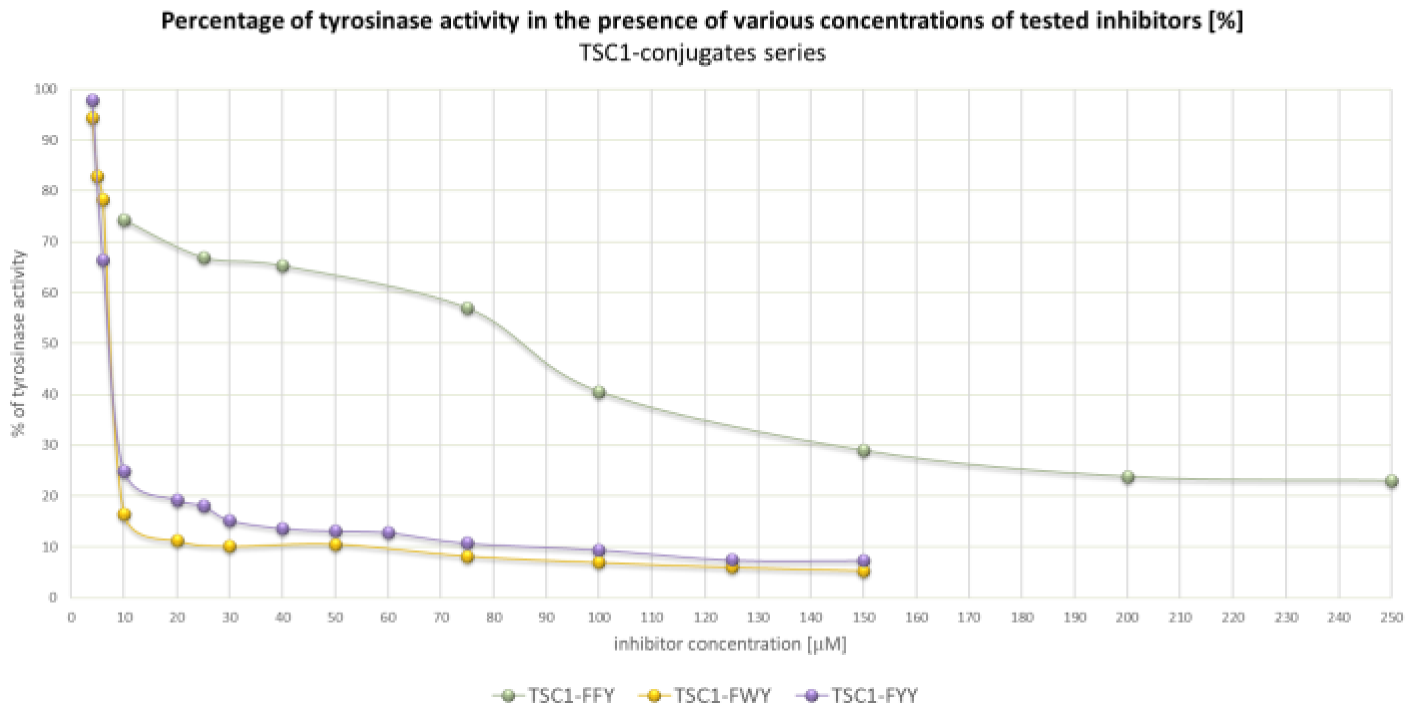

Figure SI 29. *Percentage of tyrosinase activity in the presence of compounds* ***7-9****.*

Information Classification: General

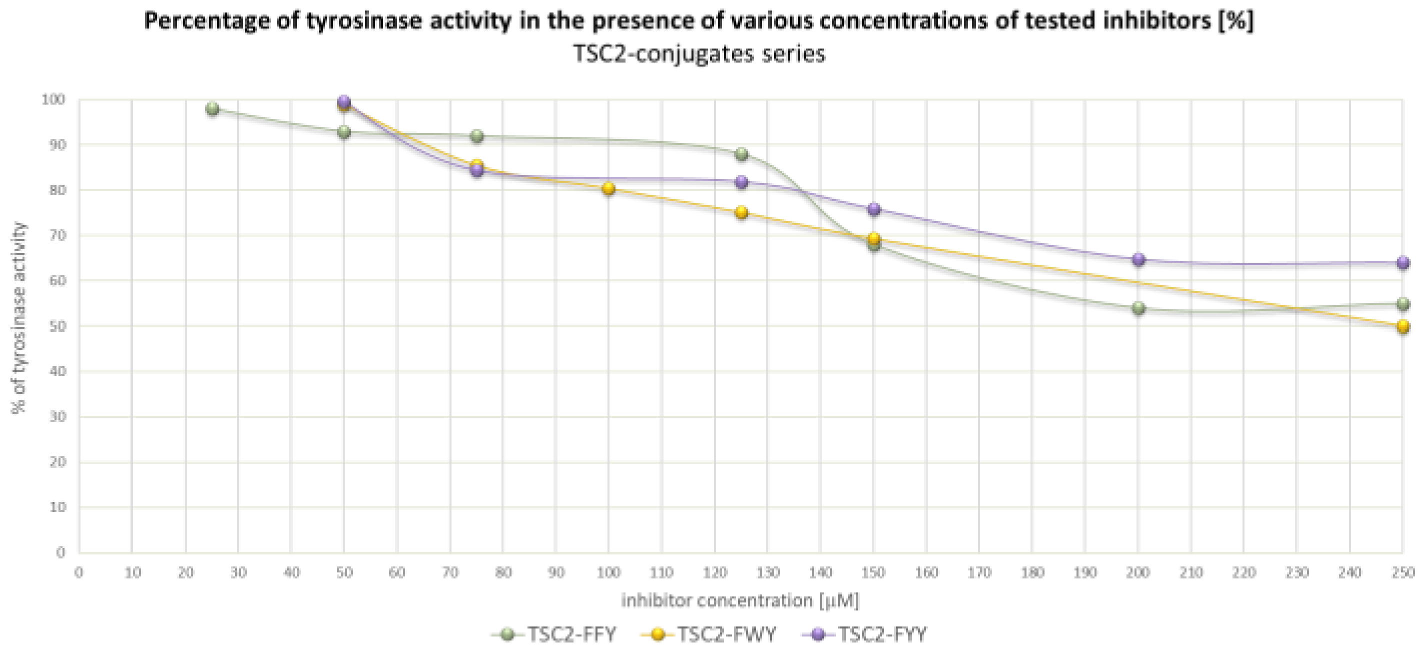

Figure SI 30. *Percentage of tyrosinase activity in the presence of compounds* ***10-12****.*

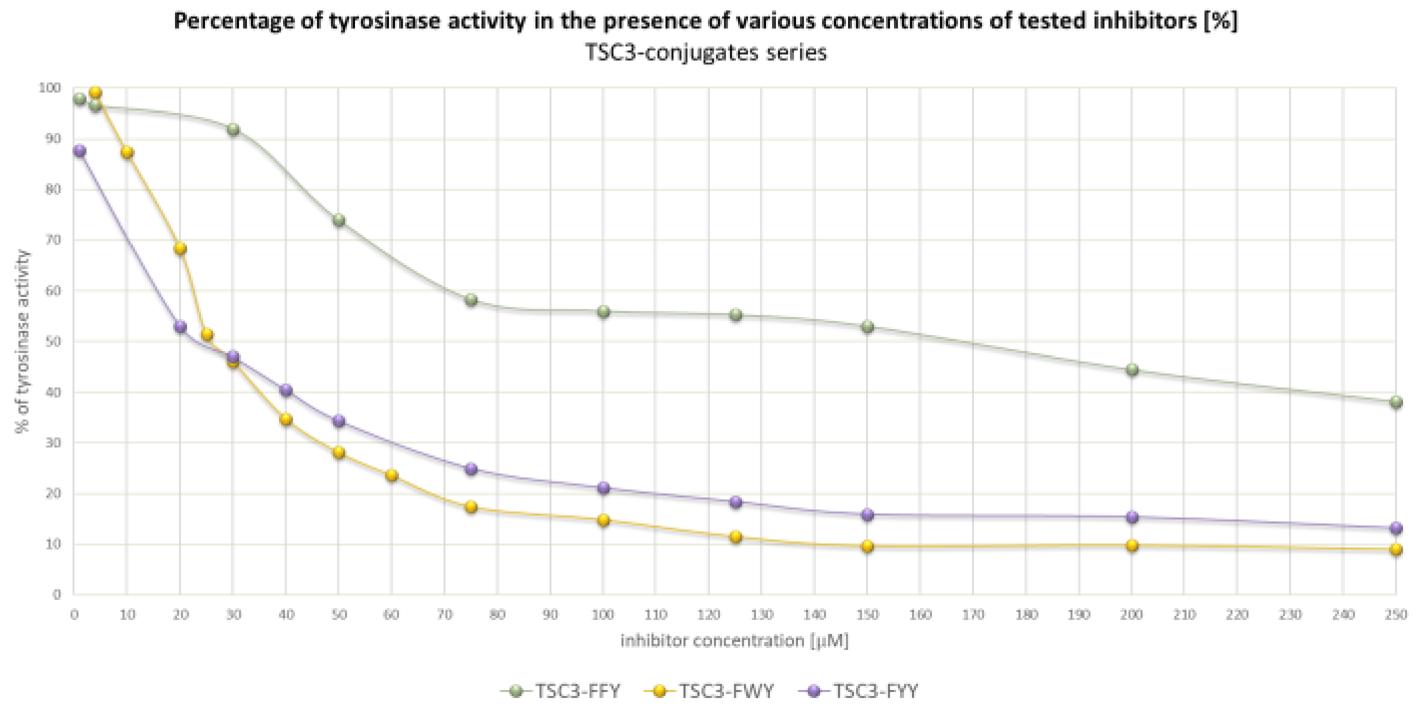

Figure SI 31. *Percentage of tyrosinase activity in the presence of compounds* ***13-15****.*

Information Classification: General

Table SI 2. *Comparison of ChemPLP scoring values for free and constrained docking protocols.*

**Inhibitor**	**ChemPLP Fitness**	
	Free docking	***Constrained docking***
TSC1	80.6	23.0
TSC2-*R*	65.7	47.1
TSC2-*S*	50.9	47.1
TSC3	82.9	33.6
TSC1-FYY	97.1	93.7
TSC1-FWY	102.0	81.0
TSC1-FFY	69.5	51.9
TSC2-*R*-FYY	66.7	46.9
TSC2-*S*-FYY	61.1	47.3
TSC2-*R*-FWY	60.3	41.3
TSC2-*S*-FWY	61.5	41.5
TSC2-*R*-FFY	62.1	51.1
TSC2-*S*-FFY	64.8	47.1
TSC3-FYY	95.8	83.0
TSC3-FWY	97.9	58.6
TSC3-FFY	78. 1	56.1
Kojic acid	53.5	

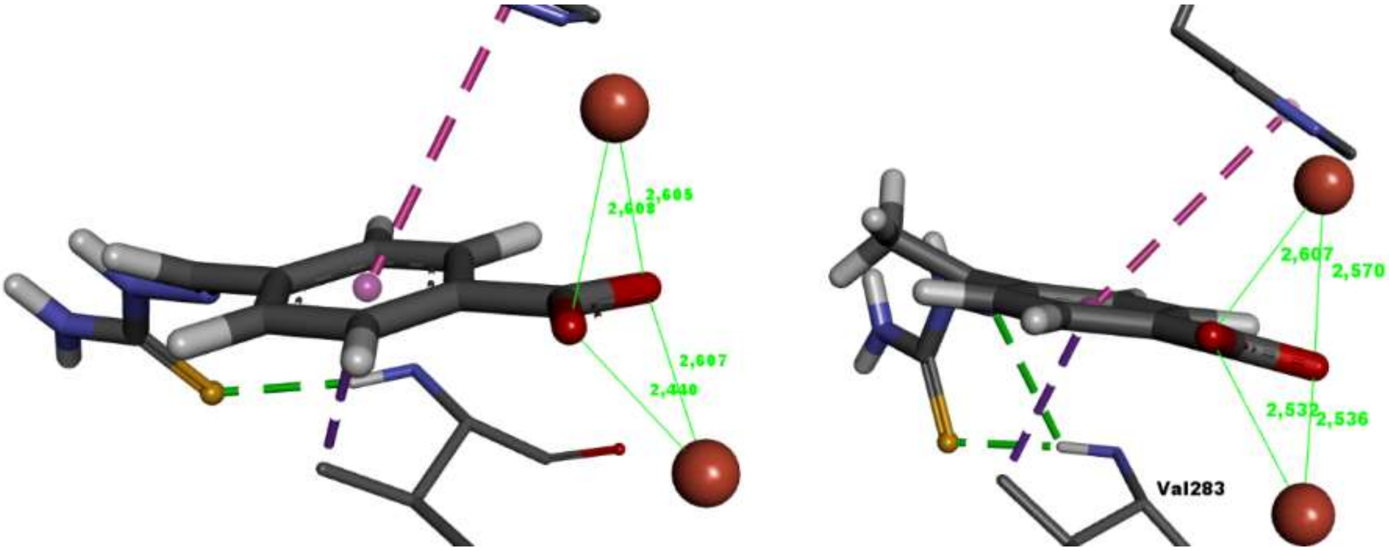

Figure SI 32. *Distance values between the carboxylic group of inhibitor and enzyme's copper ions. (TSC **1** - left, TSC **3** - right). Copper ions are shown as brown spheres.*

Information Classification: General
